# Male–Female Fertility Differentials Across 17 High-Income Countries: Insights From A New Data Resource

**DOI:** 10.1007/s10680-020-09575-9

**Published:** 2021-01-20

**Authors:** Christian Dudel, Sebastian Klüsener

**Affiliations:** 1 Max Planck Institute for Demographic Research, Konrad-Zuse-Str. 1, 18057 Rostock, Germany; 2 Federal Institute for Population Research, Wiesbaden, Germany; 3 Vytautas Magnus University, Kaunas, Lithuania

**Keywords:** Male fertility, Total fertility rate, Mean age at childbirth, Birth register data, High-income countries

## Abstract

Obtaining cross-country comparative perspectives on male fertility has long been difficult, as male fertility is usually less well registered than female fertility. Recent methodological advancements in imputing missing paternal ages at childbirth enable us to provide a new database on male fertility. This new resource covers more than 330 million live births and is based on a consistent and well-tested set of methods. These methods allow us to handle missing information on the paternal age, which is missing for roughly 10% of births. The data resource is made available in the Human Fertility Collection and allows for the first time a comparative perspective on male fertility in high-income countries using high-quality birth register data. We analyze trends in male–female fertility quantum and tempo differentials across 17 high-income countries, dating as back as far as the late 1960s for some countries, and with data available for the majority of countries from the 1980s onward. Using descriptive and counterfactual analysis methods, we find substantial variation both across countries and over time. Related to the quantum we demonstrate that disparities between male and female period fertility rates are driven to a large degree by the interplay of parental age and cohort size differences. For parental age differences at childbirth, we observe a development toward smaller disparities, except in Eastern Europe. This observation fits with expectations based on gender theories. However, variation across countries also seems to be driven by factors other than gender equality.

**Electronic supplementary material:**

The online version of this article (10.1007/s10680-020-09575-9) contains supplementary material, which is available to authorized users.

## Introduction

While the analysis of female fertility is one of the core topics of demography, fertility among men has received considerably less attention (Coleman 2000). The limited amount of comparative research on male fertility is also remarkable considering the increasing involvement of men in fertility decisions and parenting (Lappegård et al. 2011). A fact that may be surprising to some, but which has long been known in demography (Karmel 1947; Schoen 1985), is that measures of both the quantum and the tempo of fertility of males and females sometimes differ significantly in the same population. This has been observed in both high-income and low-income countries by the few existing papers that have compared male and female fertility (e.g., Nordfalk et al. 2015; Schoumaker 2019). It also holds true for the widely used fertility measures on which this paper focuses: total and cohort fertility rates and the mean age at childbirth. For instance, Schoumaker (2019) estimated that in several African countries the total fertility rate of males is up to 50% higher than the total fertility rate of females. Substantial differences between males and females have also been recorded in the mean age at childbirth. It has, for example, been shown that in many high-income countries, males are, on average, 3–4 years older at childbirth than their female partners (e.g. Nordfalk et al. 2015; UN Department of Economic and Social Affairs 2015; Dudel and Klüsener 2016). In low-income countries, even greater differences are observed. For example, according to estimates by Schoumaker (2017), fathers in Mali and Niger are, on average, up to 12 years older than mothers.

Why male and female fertility can differ substantially with respect to quantum and timing is not yet well understood. Several mechanisms have been proposed to explain differences in fertility levels, including the ratio of males to females of reproductive age (Dudel and Klüsener 2016; Keilman et al. 2014), and gender differences in the timing of births (Schoumaker 2019). But the impact of each of these and other potential mechanisms on the quantum of male and female fertility has not been thoroughly studied, and the extent of their respective contributions to fertility differentials remains unknown. The question of how the paternal age and the maternal age at childbirth develop jointly over time has also received scant attention. Moreover, there is almost no research on the question of whether men postpone fertility to the same extent as women (for an exception, see Nordfalk et al. 2015), and little is known about trends in parental age differences (for an exception, see Kolk 2015). Parental age differences have been linked to social norms and biological factors (e.g., Presser 1975; Trivers 1972), and it has also been suggested that age differences between parents might be an indicator of shifts in gender relations (Safilios-Rothschild 1972, cited by Presser 1975; Bozon 1991; Kolk 2015). Independent of age differences, advanced paternal age at childbirth has been shown to be an important predictor of the health outcomes of the offspring (Kong et al. 2012; Khandwala et al. 2017; Paavilainen et al. 2016).

Several potential explanations for the limited research on male fertility have been offered, including a lack of data and data quality issues (Poston et al. 2006). For instance, survey data on the fertility of males is generally considered to be less reliable, as males are prone to underreport their number of children (e.g., Rendall et al. 1999; Vere 2008). However, in recent years, several studies assessing male fertility based on birth register data have been published (Jalovaara et al. 2017; Bagavos and Tragaki 2017; Dudel and Klüsener 2016, UN Department of Economic and Social Affairs 2015; Nordfalk et al. 2015; Nisén et al. 2014; Carmichael 2013; Lappegård et al. 2011; Zhang 2011) that focused on single countries or on comparisons of countries. The comparative studies were, however, often limited to a cross-sectional perspective. Moreover, even though register data are usually of higher quality than survey data, they are still not free of problems, and information on the age of the father is often missing for a non-negligible share of births (Dudel and Klüsener 2018).

With this paper, we present for the first time a comparative dataset on male fertility trends for a large number of high-income countries based on birth registration data that are of high quality. Our male fertility dataset covers 17 high-income countries in North America, Europe, Asia, and Oceania (more details are available in the data section). While these data go back as far as the late 1960s for some countries, the data available for the majority of countries are from the 1980s onward. In total, the dataset contains information on more than 330 million live births. For all countries, we use birth register data, and apply a consistent and thoroughly tested set of methods to deal with data issues, including methods for imputing missing information on the age of the father. We make the age-specific fertility rates (ASFRs) underlying our analyses freely available online through the Human Fertility Collection (2019; http://www.fertilitydata.org), which accompanies the Human Fertility Database (2019). Additional materials for reproducing our findings are available through the Open Science Framework: https://osf.io/en6fk/.

Exploring the potential of this rich database, we add to the literature by studying two questions regarding male–female differentials in the quantum and the tempo of fertility. First, we analyze the ratio of the male total fertility rate (TFR) to the female TFR in order to determine to what extent male and female fertility levels differ. We also investigate the potential mechanisms behind these differences. Our analyses suggest that the degree to which male TFRs deviate from female TFRs seems to be driven by the interplay of differences between maternal and paternal ages at childbirth, combined with variation in cohort sizes. Second, we shed light on trends in parental age differences in a comparative perspective, and examine whether men postpone fertility to the same extent as women. We find that in recent years, fertility postponement has generally been more pronounced among women than among men, which has resulted in the age differences between fathers and mothers becoming smaller. This finding generally fits with gender theories about the shifting roles of women in the gender revolution (Goldscheider et al. 2015; Klesment and Van Bavel 2017). The only exception to this pattern is observed in Eastern Europe, where processes of societal restoration (Fodor and Balogh 2010) have been accompanied by increases in age differences. However, in contrast to the trends over time, the variation in age differences across countries, cultures, and welfare state types does not match expectations based on gender theories.

Before presenting our results, we first review some potential explanations and the mechanisms driving differences in the quantum and the timing of male and female fertility. We then provide an overview of our new database and its sources, and of the methods we used for preparing the data. In the outlook section, we discuss the substantial analytical potential our new male fertility database offers.

## Differences in Quantum of Male and Female Fertility

The fertility levels of males and females, as measured by the total fertility rate (TFR) or cohort fertility rate (CFR), can differ substantially, and male fertility can be both lower and higher than female fertility (Zhang 2011; Schoumaker 2017, 2019). From a demographic perspective, such disparities can be driven by three main factors: first, differences in the population sizes of males and females, i.e., in the sex ratio, which can affect both TFR and CFR differences; second, age differences between mothers and fathers in interplay with variation in cohort sizes (affecting the TFR and the CFR); and, third, gender differences in tempo effects, which can impact gender differences in the TFR, but not in the CFR.

Uneven sex ratios among females and males of reproductive age can emerge as a result of different processes. One potential contributor to these differences is the sex ratio at birth. For many populations, the sex ratio at birth in non-crisis situations is at around 105 males per 100 females (Markle 1974; Lazarus 2002). To see how this can affect fertility levels, consider a simple hypothetical scenario that ignores mortality, and in which there is only one cohort of males and females having children with partners of the same cohort. As the number of births would be the same for males and females, the numerator of the average number of children would also be the same. But the denominators would differ because of the sex ratio at birth, and the average number of children among males would be lower by a factor of 100/105.

Two other factors shaping sex ratios are differential mortality and sex-selective migration patterns. Low male-to-female sex ratios in the general population due to male survival disadvantages can occur as the result of wars (Dinkel and Milenovic 1993) or a higher tendency among males to engage in health adverse behavior. An extreme example of the latter factor is the high premature male mortality in Eastern Europe due to alcohol consumption (Leon et al. 2009; Grigoriev et al. 2015). As migration patterns are often sex-selective, they can also affect sex ratios. In eastern Germany, for example, women were more likely than men to leave the region for a period of time following German reunification, which resulted in high male-to-female sex ratios in the population of reproductive age (Kröhnert and Vollmer 2012).

Age differences between mothers and fathers can affect male–female differences in fertility when there is variation in cohort sizes (Schoen 1985). Generally, the reproductive age range of men is longer than that of women (Dudel and Klüsener 2016); and fathers are, on average, older than mothers (e.g., Kenrick and Keefe 1992). If mothers and fathers come from different birth cohorts and there is variation in cohort size, the denominators of male and female fertility rates will differ. For instance, Schoumaker (2017) has shown that the rapid expansion of African populations can lead to male TFR levels increasing more than female TFR levels, as fathers tend to come from the older and smaller cohorts (see also Field et al. 2016). A related pattern with potential implications for male/female differences in fertility outcomes is the transition from the baby boomer to the baby bust generation, which many high-income countries experienced in the mid-twenty century. Such a population trend might cause male fertility to decrease relative to female fertility, as fathers are more likely to belong to the older, bigger cohorts.

Tempo effects might also contribute to gender differences in fertility, at least for period-based measures like the TFR. Period measures are affected not only by changes in the quantum of births, but also by changes in the timing of births (tempo effects), as has been extensively demonstrated for females in the context of fertility postponement and delayed childbearing (e.g., Kohler et al. 2002; see the next section for a more detailed discussion). If these tempo effects differ between males and females, then period-based measures of fertility levels will differ accordingly.

## Differences in the Timing of Male and Female Fertility

### Why are Fathers Older on Average? Social Status and Limiting Factors

Within human populations, fathers are, on average, older than mothers. This pattern seems to hold across time and space (Kenrick and Keefe 1992; Schoumaker 2017, 2019). For instance, in high-income countries, males are, on average, 2 to 4 years older at childbirth than their female partners (Kolk 2015; Dudel and Klüsener 2016). Intertwined social and biological perspectives have been offered to explain why fathers tend to be older than mothers.

From a social status perspective, it can be argued that women have, at least historically, had fewer opportunities than men to acquire social status, be it through education or work (Presser 1975). Thus, it was common for a woman to secure her social status through a partnership with a man (Blake 1974, cited by Presser 1975). A related point is that a man who is older than his female partner is likely to be at an advantage compared to a man who is the same age as the woman, as an older man is likely to be more established in his life, which reduces the couple’s uncertainty about their future social status trajectory (Presser 1975). This dynamic has probably contributed to the emergence of a pattern in which a woman is less likely to partner with a younger man (Bozon 1991). Moreover, especially in societies with a strong patriarchal orientation, social norms regarding male and female reproductive behavior have been instrumented to stabilize existing sex- and age-related social inequalities (see, e.g., Szołtysek et al. 2017).

Proponents of an evolutionary perspective of parental investment (Trivers 1972) have argued that both females and males have to be selective in their partner choices, as among humans, it is usually necessary for both partners to invest heavily in their offspring (Kenrick and Keefe 1992). However, while the investments of females are high in terms of bodily resources (pregnancy and lactation), the investments of males are more indirect (providing resources such as food or security). Based on this perspective, it has been argued that when choosing a partner, females are more focused on whether the male is able to provide indirect resources, while males are more focused on whether the female is healthy and has reproductive potential (Kenrick and Keefe 1992). Among men, the ability to provide resources—as, for example, signified by the man’s social status—generally increases with age, which tends to put older males at an advantage. The reproductive potential of females, on the other hand, decreases with age. These two mechanisms could also explain why the male partner in a couple is often older than the female partner.

However, from a theoretical point of view, it is also possible to identify mechanisms that could lead to partners having more similar ages. It has been shown that the relationships of partners with shared characteristics tend to be of higher quality (e.g., Gaunt 2006). Age homogamy not only increases the likelihood of having similar attitudes, it decreases the likelihood of unequal power relations emerging (see Dribe and Stanfors 2017). Thus, under conditions in which there are no strong factors favoring age heterogamy in couples, many individuals might prefer to partner with a person of a similar age, as doing so may increase the quality and the durability of the relationship. Moreover, having a stable relationship could, in turn, have implications for the couple’s ability to support the development of their potential offspring (see also Kenrick and Keefe 1992).

### The Gender Revolution, Fertility Postponement, and Changes in Age Differences

While fathers are, on average, older than mothers, there has been considerable heterogeneity in these age differences across time and space. Historical research on the age at marriage has shown for a number of European countries that age differences within couples were larger in the 19th and the early twenty century than they were in recent years (Presser 1975; van Poppel et al. 2001). Several partly related factors might have caused age differences to decline in high-income countries. These include the establishment of the modern welfare state, the gender revolution (Goldscheider et al. 2015), and differential levels of postponement of fertility.

The rise of the modern welfare state has likely contributed to the decrease in the relevance of a set of factors that would otherwise tend to encourage greater age differences within couples. Having access to welfare state benefits can make a woman less dependent on her male partner as a provider of support (Esping-Andersen 1999; McLaughlin and Glendinning 1994). Moreover, modern health care systems and reproductive technologies have led to a decrease in the relevance of natural fecundity over the life course for successful reproduction–even though assisted attempts to have a child do not always result in a birth (Gameiro and Finnigan 2017; Leridon 2004). Under these circumstances, age heterogamy might be less advantageous, which could lead to a reduction in the age differences between partners.

The gender revolution has substantially increased women’s opportunities to obtain social status through their own education and work (Goldscheider et al. 2015). Meanwhile, female enrolment in higher education has risen considerably, surpassing that of men in many countries (Grow et al. 2017). This trend toward gender equality might affect age differences in two ways. First, tertiary education extends well into early adulthood and the ages at which family formation typically occurs, while also bringing together individuals in a rather limited age range, which could lead to increased homogamy (Kolk 2015). Second, as it becomes increasingly difficult for a woman to find a partner with similar or higher educational attainment, she may be willing to marry a man with a lower educational level (Esteve et al. 2016; Klesment and Van Bavel 2017). If women are accepting male partners with lower educational attainment, these men might also be younger than potential partners with similar or higher educational attainment.

Differential patterns of postponement of fertility to older ages is a mechanism that could affect differences in the mean paternal and maternal ages at childbirth. If, for instance, postponement was more pronounced among females than among males, the mean age at childbirth would increase faster for females than for males. While fertility postponement among females has received considerable attention in the demographic literature (Kohler et al. 2002; Sobotka 2004; Fox et al. 2018), this has not been the case for males (Nordfalk et al. 2015). A potential mechanism for gender differences in postponement could be increased educational hypergamy, as described above. If women who are highly educated tend to postpone childbirth and have children with partners who have similar or lower educational levels and are of similar ages, postponement among women would not necessarily translate into a similar degree of postponement among males.

## Data and Methods

### Data

To investigate how and why male and female fertility differ, we created a large, novel database on male fertility. We make the database available as part of the Human Fertility Collection. In this database, we provide annual age-specific fertility rates (ASFRs) for men based on counts of live births for 17 high-income countries. Countries were included based on two criteria. First, we tried to include countries from each of a total of six groups or types covered by the welfare state literature: the three original types proposed by Esping-Andersen (1990; corporatist, social-democratic, liberal), as well as the Mediterranean type (Arts and Gelissen 2002), the Central/Eastern European type (Fenger 2007), and the East Asian type (Aspalter 2006). The second criterion was data availability, which in this case means both that information on men exists in the national birth register data, and that these data can be accessed.

An overview of the data is provided in Table 1. It shows for each country the years covered, the total number of births on which the ASFRs are based, and the smallest and the largest annual proportion of births with unknown age of the father (see below for a discussion). For a complete description of the data and the underlying methods, see Dudel and Klüsener (2019).

**Table 1:** Overview of covered countries and years

Country	Years	# births	Min. % missing	Max % missing	Splitting age intervals?
Australia	1975–2014	15,802,285	< 1	<1	No
Canada	1974–2011	10,312,460	4	11	No
Denmark	1986–2015	1,899,172	10	47	No
England & Wales	1982–2016	23,345,243	5	8	Yes
Estonia	1989–2014	391,541	5	17	No
Finland	1987–2015	1,741,444	1	4	No
France	1998–2013	12,507,805	–	–	Yes
Germany	1991–2013	20,905,142	7	22	Yes
Hungary	1970–2014	5,531,052	2	14	No
Italy	1999–2014	8,743,545	5	11	No
Japan	2009–2016	6,262,714	2	2	Yes
Poland	1986–2014	12,735,720	3	5	No
Portugal	1980–2015	4,128,924	2	5	Yes
Spain	1975–2014	18,572,616	1	3	No
Sweden	1968–2015	5,010,338	1	5	Yes
Taiwan	1998–2014	3,820,183	< 1	< 1	Yes
USA	1969–2015	179,487,897	8	23	No

# births relates to the cumulative number of births covered for each country; Min.  % missing and Max  % missing to the smallest and the largest annual proportion of births with missing paternal age. For the French birth register data, missing paternal ages were imputed by the French Statistical office; thus, the raw data we used did not show any missing values. The last column (splitting age intervals) indicates whether five-year age intervals and/or open-ended age intervals had to be split. See Sect. 4.2 for details

The data were obtained from the national statistical offices, and the US data were provided by the National Bureau of Economic Research. All of the birth counts were derived from birth registers and were based on complete enumeration, except for the USA for the years from 1969 to 1984. For Taiwan and Italy additional information from the Human Fertility Database (2019) was used (see Dudel and Klüsener 2019 for details). Population exposure data was taken from the Human Mortality Database (2019). The raw data for western, eastern, and total Germany were obtained from data collected by Dudel and Klüsener (2016), with some minor modifications described in Dudel and Klüsener (2019).

For all countries, we provide data by single-year ages for the age range of 15 to 59 years. We chose 59 as the highest age, as fathers aged 59 or older are rare. The highest proportion of fathers aged 59 + across all of our countries and years was 0.2% in Italy in 1999, and the proportion was considerably lower for most other countries and years. Generally, it can be observed that the age-specific fertility rates of men quickly decline to very low levels above age 45, at least in high-income countries (see also Nisén et al. 2014; Nordfalk et al. 2015). Thus, while a share of male fertility occurs at higher ages, it appears that male fertility is mostly concentrated over an age range of roughly 25 years (age 20 to age 45)—despite claims to the contrary sometimes made in the literature (see Poston et al. 2006 for a discussion). This observation can be explained by decreasing male fecundity and the declining fecundity of the female partner (who is, on average, 2 to 4 years younger than her male partner), as well as age norms regarding childbearing (Billari et al. 2011).

### Methods for Data Preparation

As Dudel and Klüsener (2018) pointed out, one of the biggest challenges researchers face when studying male fertility are missing values for the age of the father, as in many datasets, the paternal age is not recorded for a sizable number of births. For the countries and the years we study, the proportion of missing values ranges from below 1% (e.g., Sweden, 2002) to 47% (Denmark, 1994). For some countries, the proportion also varies considerably over time. For instance, in Germany, the highest proportion of missing values was recorded in 1999, at 22%; but the proportion had decreased to 7% by 2013.

Missing values might be due to the mother not knowing the father, or not wanting to provide information on the father. Depending on the country and the year, other factors may also play an important role. For instance, in Germany until 1999, the paternal age was recorded for marital births only, and not for non-marital births (Dudel and Klüsener 2016). Conversely, in Sweden, the proportion of missing values has generally been very low, at around 1%; but was around 5% in 1998. This pattern can be explained by procedural changes in Sweden that led to the statistics for 1998 being derived from the birth register very early in 1999. At that time, paternal ages were still missing for a considerable share of non-marital births, as there is a fixed period after the initial registration during which the mother can add missing required information on the father (Statistics Sweden, personal correspondence, February 16, 2018). Among the factors that might lead to missing information on the father are fertility within same-sex couples and “fatherless” reproduction due to assisted reproductive technologies. However, as the number of such births is currently relatively low, we assume for our calculations that all births have a mother and a father. In 2010, for instance, the proportion of births attributable to all assisted reproductive technologies was between 1 and 2% in the countries we study (Dyer et al. 2016).

In this paper, we use the conditional imputation approach, which is generally superior to other approaches, such as the unconditional approach, as demonstrated by Dudel and Klüsener (2018). This approach requires that both the paternal and the maternal age are known for some births, and it imputes the missing paternal ages conditional on the age of the mother. The intuition underlying this approach is that the age of the mother is an excellent predictor of the paternal age and thus should be included when imputing the paternal age. Here, we explain this approach using a simple case in which the maternal age is recorded for all births, while the paternal age is missing for some births, in the birth register data of a given country and year. For each maternal age (e.g., 30) this approach starts from the number of births for which the age of the father is missing. To impute the paternal ages for these births, the conditional age distributions of fathers, assuming that the mother is aged 30, are calculated. The births to mothers aged 30 and the missing paternal ages are then assigned paternal ages according to this distribution. If, for example, there are 1000 births with a maternal age of 30 and a missing paternal age, and 10% of the births to mothers aged 30 with a known paternal age are to fathers aged 33, then 10%, or 100, of the births with a missing paternal age will be assigned a paternal age of 33 years. For a more detailed discussion, see Dudel and Klüsener (2019).

For some countries, additional methods were applied. This includes the penalized composite link model (PCLM) of Rizzi et al. (2015), as implemented by Pascariu et al. (2018), to split open-ended age intervals into single ages (England/Wales, Portugal, France, Sweden), and to split 5-year age groups into 1-year intervals (Portugal, Taiwan). For Sweden the data provides the parental ages not at the time of childbirth, but at the end of the year in which the child was born. To calculate the parental ages at childbirth based on this data, we applied an interpolation method used for this purpose in the HFD (Jasilioniene et al. 2015). For Germany and Sweden, the youngest available age in the data is 17 and not 15. We split the interval covering age 17 and under by distributing the births to ages 15, 16, and 17 based on the distributions of births for these ages observed for the other countries. More detailed discussions of all these methods can be found in Dudel and Klüsener (2019).

To compare male and female fertility, we also derived ASFRs for women using the same data sources as those we used for men. Thus, the ASFRs for men and women are based on the same underlying data. The ASFRs for women are available as part of the replication materials on the Open Science Framework. The differences between the female ASFRs derived from our data and those obtained from other data sources like the Human Fertility Database (HFD) are small; thus, using the ASFRs for women derived from the HFD would essentially lead to the same results. For women, we cover the age range between 15 and 49 years.

### Analytical Strategy and Main Questions

Our analysis will focus on two main questions. First, we will present new findings on trends in quantum differences for 17 high-income countries, showing how the male total fertility rate (TFR) and the female TFR differ. An intuitive and commonly used way to compare male and female fertility levels is to calculate the ratio of the male TFR to the female TFR (e.g., Schoumaker 2019; Keilman et al. 2014). If this ratio is equal to one, the period fertility levels of men and women are the same; if it is higher than one, male fertility is higher; and if it is below one, male fertility is lower. To disentangle the factors driving the differences, we will also present results on the cohort fertility rate (CFR), which, unlike the TFR, is not affected by tempo effects (Ni Bhrolchain 1992). As the calculation of CFRs requires long time series of data, we can study these rates for a few countries only: namely, Australia, Canada, Hungary, Spain, Sweden, and the USA. In addition, we will perform for all countries a counterfactual analysis in which we will attempt to disentangle deviations stemming from age differences in couples from deviations resulting from variations in the sex ratios of males and females.

Our second question focuses on trends in gender differences in fertility timing: to what extent have age differences between mothers and fathers been diminishing over time? This question is based on the theoretical considerations discussed above which posit that increasing gender equality over time might lead to smaller age differences. To address this issue, we focus on differences in the mean age at childbirth between men and women measured in years; an easily interpretable outcome that has been used in the previous literature (e.g., Kolk 2015). We also seek to determine whether more gender-equal countries are indeed at the forefront of this trend.

For the analyses we group our countries using welfare state typologies (Esping-Andersen 1990; Arts and Gelissen 2002; Aspalter 2006; Fenger 2007) as an orientation: the social democratic Nordic countries (Denmark, Finland, Sweden); the corporatist Western European countries (France; Germany); the—at least historically—rather conservative Southern European countries (Italy, Portugal, Spain); the former communist countries of Eastern Europe (Estonia, Hungary, Poland); the liberal and predominantly English-speaking countries (Australia, Canada, England and Wales, United States of America); and the East Asian countries (Japan, Taiwan).

## Results

### Male–Female Disparities in Fertility Levels

In all of the countries, the male TFR trend generally follows the female TFR trend (detailed results for all countries are shown in the appendix, Figure A1-A17). In Sweden, for instance, the male TFR exhibits the same “rollercoaster” pattern (Hoem 2005) as the female TFR. The Swedish male TFR is, however, lower for almost all years, except for the first two observation years (1968 and 1969). Moreover, the male TFR in Sweden is slightly more volatile than the female TFR; a finding that holds for the majority of the countries we study.

**Fig. 1:**
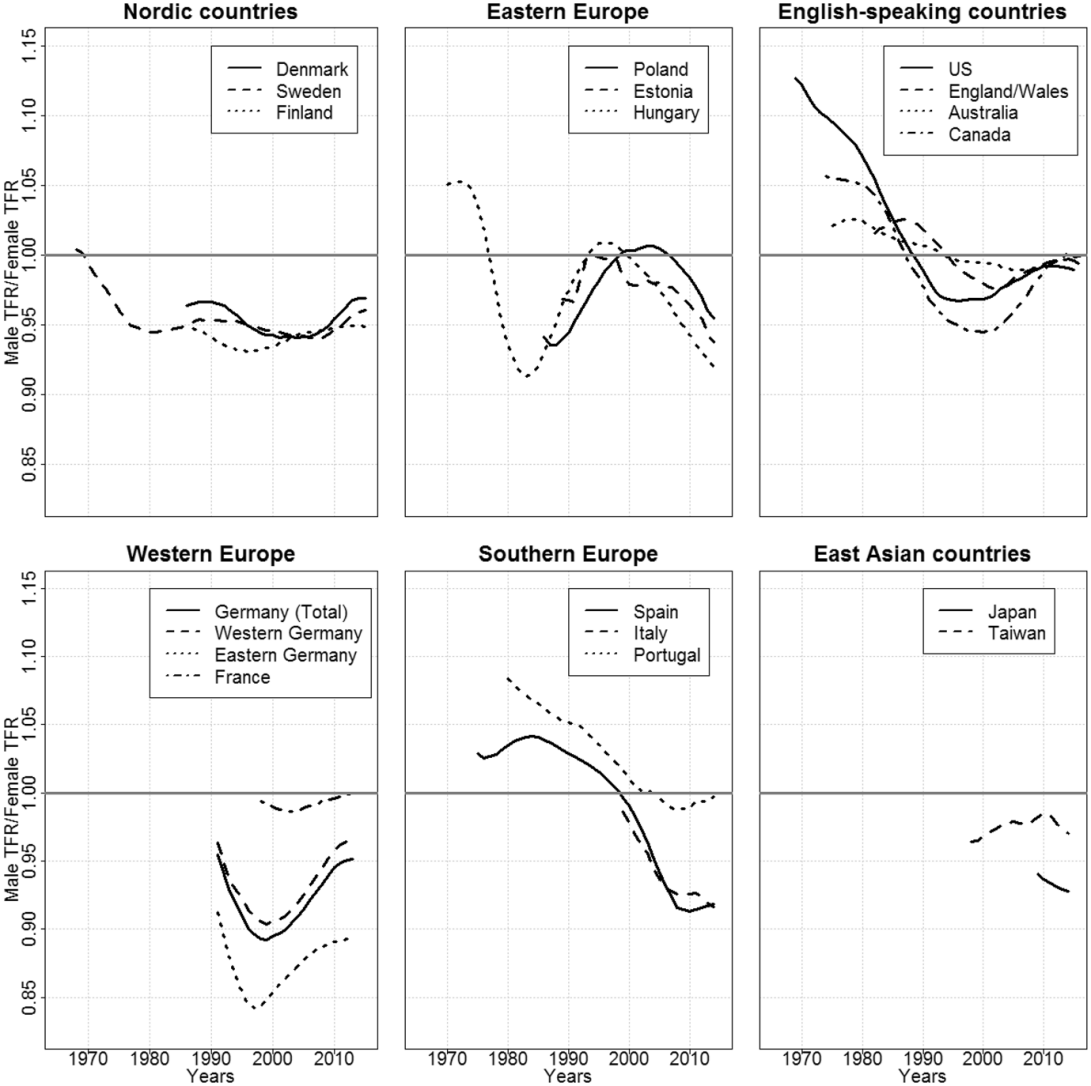
Male/Female TFR ratios by country group. Source: Own calculations

In Fig. 1, we report for each country the trend in the ratio of the male to the female TFR, arranged by country group. In the set of countries and years we cover, the ratio of the male TFR to the female TFR ranges from 0.84 (eastern Germany, 1997) to 1.13 (the USA, 1969). Thus, while male and female fertility levels, as measured by the TFR, appear to be highly correlated, we observe no clear one-to-one relationship between them (see also Karmel 1947). Moreover, there seems to be a tendency for ratios to shift from higher to lower levels over time.

The trends within each of our country groups are mostly consistent. In the Nordic countries we cover, the male fertility level has been relatively constant for the last 30 years at around 95% of the female fertility level. In the Eastern European countries, the ratio follows a hump-shaped curve: i.e., there was an initial period of decline, which was then followed by an increase and another decline. A surge in the ratio occurred in all three Eastern European countries at the end of the communist period and in the 1990s, and was followed by a decline after 2000. We will discuss potential explanations for these patterns below. In all three Southern European countries, the TFR ratio dropped considerably. In Portugal and Spain, for which we have longer time series than for Italy, the ratio was above one at the beginning of the observation period. But by the end of the period, the ratio was below one in all three countries, with Italy and Spain displaying particularly low values, at around 0.92. While the pattern in the English-speaking countries is less consistent, we can see that in all four countries the ratio dropped from above one to below one, and then recently increased slightly to reach values of around one. In the Western European and the East Asian countries, no clear patterns emerge based on the available data. It is, however, interesting to note that in Germany since 1990, post-communist eastern Germany has followed a U-shaped pattern similar to that of western Germany, but at lower levels. This trend diverges from the hump-shaped patterns we obtained for this period for the post-communist countries of Estonia, Hungary, and Poland.

### A Cohort Perspective on Level Differences

As the TFR is affected by tempo effects, it is also of interest to investigate whether and, if so, to what degree differences vary if cohort fertility rates are studied. The time series for a small number of the countries in our sample are long enough to obtain cohort data for at least some cohorts, if we restrict the age range to 15 to 50. These cohort data are derived from period ASFRs based on the assumption that the in- and out-migration levels of men are not selective given their achieved fertility. Our cohort analysis is mainly focused on Sweden, the USA, and Hungary, as cohort data is available for only a few or for no cohorts in the other countries. However, we also show results for Canada, Australia, and Spain, as we have relatively long series for these countries as well.

**Fig. 2:**
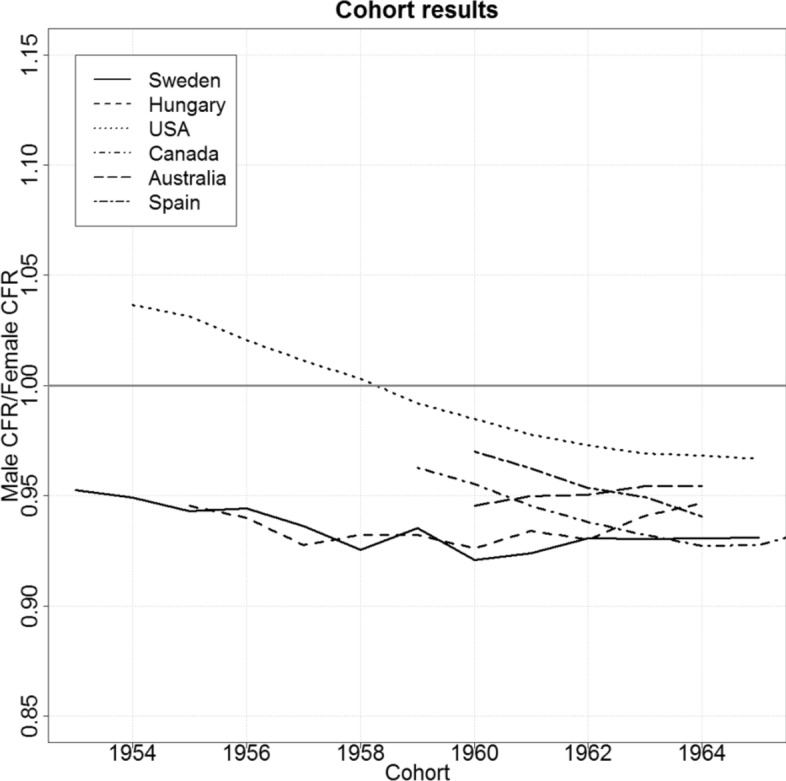
Male/Female Ratio of the Cohort Fertility Rate for Sweden, Hungary, and the US Source: Own calculations

The results are shown in Fig. 2. For Sweden, the cohort patterns are very similar to the period patterns. For each cohort, the average number of children is 5% lower among males than among females. In Hungary, the rollercoaster pattern observed in the period perspective (see Fig. 1) is not visible in the cohort pattern, at least for the birth cohorts we are able to cover (1955 to 1964). The ratio for Hungary is roughly the same as the ratio for Sweden. In the USA, male fertility was higher than female fertility among the older cohorts (1954 cohort: 104%), whereas female fertility was higher than male fertility among the younger cohorts (e.g., 1965: 97%). While these cohort patterns are generally similar to the TFR trends in the USA, the differences between male and female fertility are less extreme.

Interestingly, the cohort pattern in the USA is in line with the mechanism underlying the “birth squeeze” hypothesis of Schoen (1985), according to which a move from bigger to smaller cohorts might put males at a disadvantage. This is due to the above-described pattern that in many (childbearing) couples the male partner tends to be older than the female partner (Presser 1975; Schoen 1985; Dudel et al. 2020), who come from smaller cohorts. US birth rates started to fall after 1959, and the 1959 birth cohort is also approximately the cohort in which we observe a shift from a positive to a negative male/female fertility ratio. However, when we also take other countries in account, the evidence for a “birth squeeze” is rather mixed. For example, in Australia, which experienced the end of the baby boom at a similar point in time as the USA, the ratio was increasing slightly among the cohorts born in the 1960s. Another counter-example is Spain, which did not experience the end of the baby boom until the 1970s, but was still witnessing a declining male–female fertility ratio among the cohorts born in the 1960s.

Overall, the flat CFR ratio for Hungary suggests that the distinctive rollercoaster pattern of the Hungarian TFR ratio might largely stem from differences in paternal and maternal age and in postponement patterns. We can also see that the TFR ratio trend in the USA was likely also affected by these factors, while in Sweden the cohort and the period ratios hardly differ.

### Counterfactual Assessment of Period Fertility

To further disentangle the determinants of disparities between male and female fertility for a larger set of countries, we derive male fertility rates for a hypothetical scenario in which all women have their children with men of the same age. Thus, we are left with just the differences that stem from sex ratio differences in each cohort of potential mothers and fathers. To achieve this we erase the paternal age information for all births. Then we assume for each birth that the mother’s age (e.g., 25) is the same as the father’s age. As a result, differences in the reproductive age range, tempo distortions due to postponement, and age differences within couples can no longer affect the fertility differences between males and females.

**Fig. 3:**
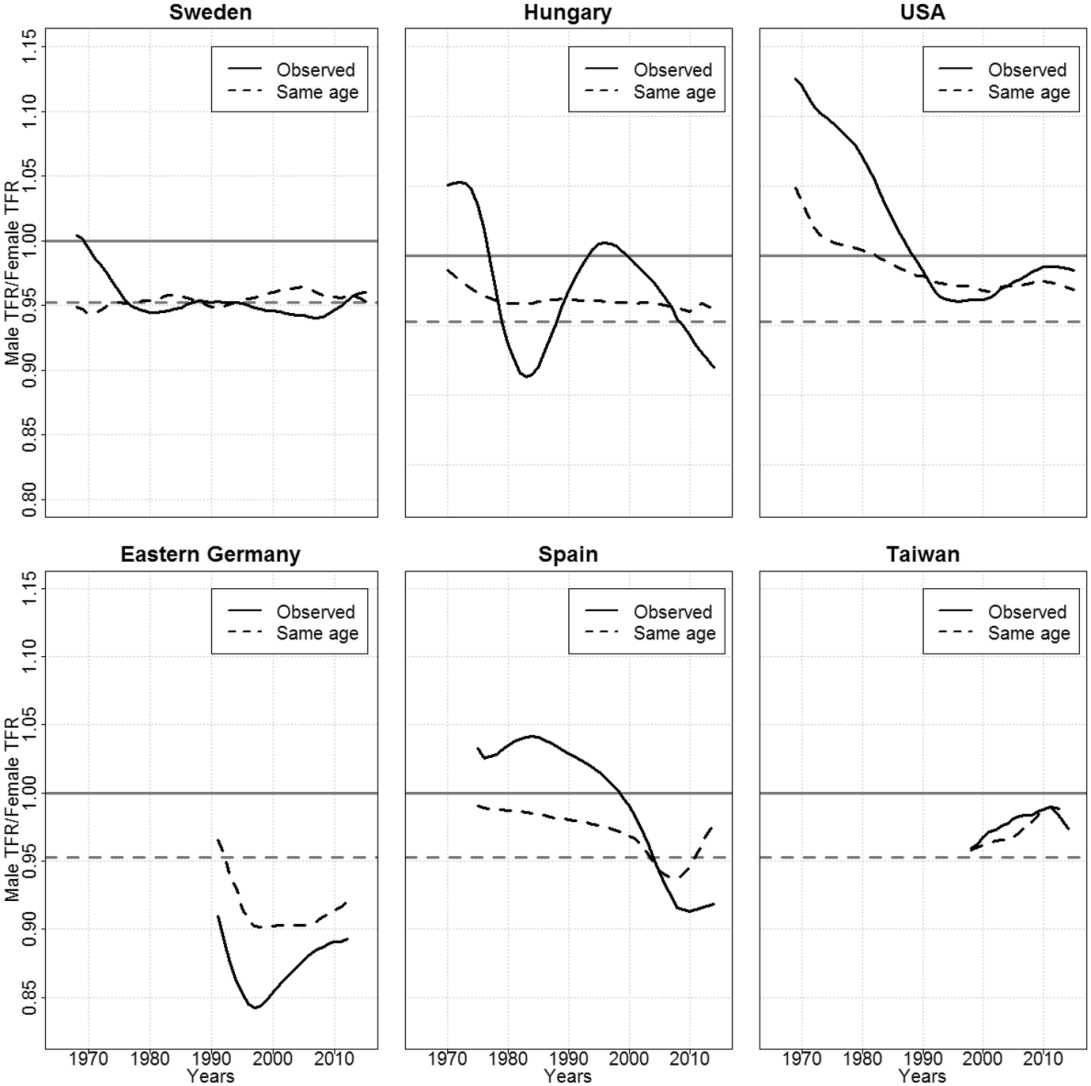
Hypothetical Male/Female TFR ratios in which fathers are assigned the maternal age at birth by country group. Source: Own calculations

Selected resulting graphs are presented in Fig. 3, which covers one country of each country group; while the complete set of graphs are provided in Figures B1-B17 in the appendix. For orientation, we added a line slightly above 0.95, which would be the TFR ratio if the sex ratio at birth was 105 males to 100 females, and if there was no sex-selective mortality and migration until the end of the reproductive lifespan. If the counterfactual TFR ratio is above or below this line, fertility differences between males and females would likely be driven by imbalanced sex ratios due to migration and/or mortality. Specifically, if the sex ratio at birth was indeed 105 males to 100 females, then a counterfactual TFR ratio above the line would be indicative of excess females due to migration (e.g., more women immigrating than men) and/or mortality (females having higher survival rates); and vice versa. The effects of migration and mortality might also go in the opposite direction (e.g., more men immigrating, but women having higher survival rates), and could even cancel each other out. Figure 3 also shows the observed TFR ratios. If the observed and the counterfactual TFR ratios differ, we can assume that, in addition to migration and mortality, the age differences between mothers and fathers play a role in the fertility differences between males and females.

The counterfactual results for Sweden, Hungary, and the USA are close to the results for the CFR ratios. Specifically, for Sweden, the counterfactual differences in male and female fertility are close to the observed ratio, as the latter does not seem to be affected by age differences (the dashed line and the solid line are close), nor to be distorted by imbalanced sex ratios due to migration or mortality (the dashed line is on the gray dotted line, slightly above 0.95). The pattern for Hungary seems to be primarily the result of differences in the timing of fertility (the dashed line and the dotted line are close, while the solid line differs considerably), although a minor influence of migration and mortality is visible. Looking at the USA, we can see that differences in sex ratios likely caused differences in fertility levels, at least before 1990 (the dashed line is getting closer to the dotted line over time); and that age differences had an additional effect (the solid line is above the dashed line before 1990).

For Spain, it appears that age differentials contributed to differences in fertility levels. No such pattern is observed for Taiwan. Interestingly, eastern Germany is the only example shown here with a counterfactual TFR ratio that is mostly below 0.95, and is thus below the value that would arise if there were no imbalances in the sex ratio due to migration and mortality. As female mortality was considerably lower than male mortality in eastern Germany (Kühn et al. 2019), this leaves migration as the likely cause. Indeed, females have been more likely to leave eastern Germany than males, which has led to an imbalanced sex ratio (Kröhnert and Vollmer 2012). It has been speculated that this imbalance could have contributed to the rather low male fertility levels in eastern Germany (Dudel and Klüsener 2016). Overall, our counterfactual analysis suggests that most of the observed temporal variation in TFR ratios can be related to age differences between fathers and mothers who originated from cohorts of different sizes.

### Trends in Timing of Births

To assess differences in the timing of births, we look at the mean age difference between fathers and mothers. If this difference is positive, males are, on average, older than females. The results are shown in Fig. 4 (detailed results are available in the supplementary materials, Figures C1-C17).

**Fig. 4:**
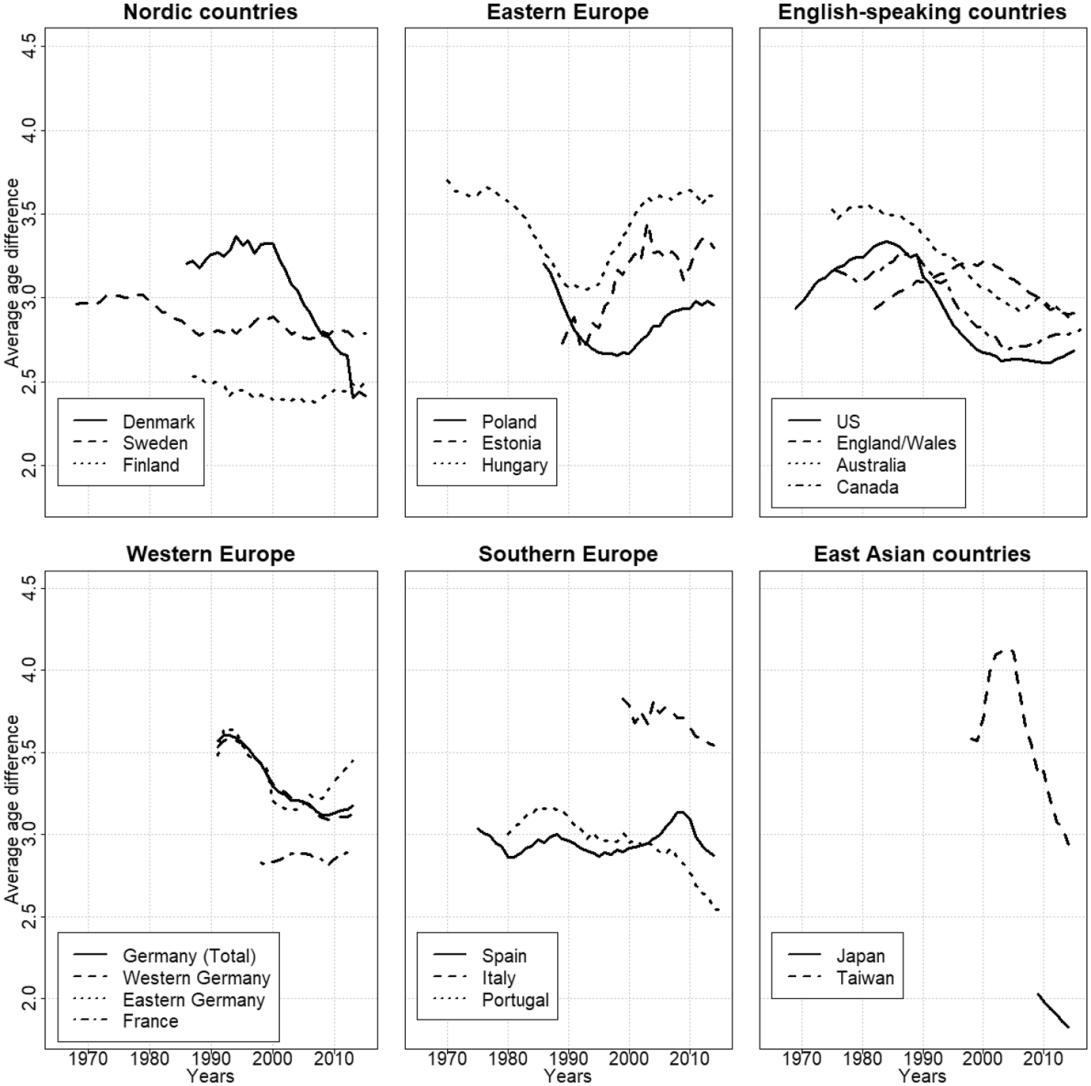
Trends in the average age difference between the father and the mother. Source: Own calculations

When we look at the differences in the mean age at childbirth, we can see that the similarities within our country groups are less visible compared to the outcomes for the TFR ratios. This is perhaps most visible in East Asia. Taiwan in the early 2000s had the largest age differences among our observed countries and periods, with fathers being, on average, 4 years older than mothers. Meanwhile, the few data points we have for Japan show that the country had the smallest age differences in our dataset, at 2 years. The volatility of the pattern in Taiwan also stands out: in the late 1990s/early 2000s, the average age difference in that country increased from 3.5 years to more than 4 years, and then fell back to 3 years in 2014.

Turning to the Nordic countries, we can see that in Sweden, the mean age difference was close to 3 years in the late 1960s, and stayed at around that level up to 1980. It declined to 2.8 years between 1980 and 1990, and has been close to that value ever since. But in the other Nordic countries, we detect no coherent pattern for age differences, unlike for differences in fertility levels. While all of the Nordic countries had smaller age differences at the end of the observation period, the trend patterns varied. In Sweden and Finland, the changes were rather smooth. This was not the case in Denmark, which had a much larger age gap than the other two countries before 2000. In 2000, the mean age difference in Denmark was around 3.3 years and almost 1 year higher than that in Finland (app. 2.4 years). This changed swiftly after 2000, as Denmark experienced a drastic decline in the mean parental age difference at birth. Thus, Denmark, together with Finland, currently has the smallest mean age difference among all of the observed European countries, at around 2.5 years.

When we look at Southern Europe, we also see substantial variation. Italy currently has among the three countries the largest mean age difference, at more than 3.5 years; but the gap in that country has been declining. Spain and Portugal currently report gaps of 3 or respectively 2.5 years. The age gap in Spain today is only slightly smaller than the gap recorded in the late 1970s. It had increased prior to the 2008 economic crisis, and then declined after the onset of the crisis. The pattern in Portugal, on the other hand, has diverged from that in Spain since the early 2000s, with the overall age gap decreasing by more than half a year since the 1990s.

Looking at the (predominately) English-speaking countries, we can see that the neighboring countries of Canada and the USA display marked similarities in age difference levels and trend patterns. In these countries, age disparities increased slightly in the 1970s and 1980s, to around 3.25 years; and then declined in the 1990s, leveling off at around 2.6 years. In Australia, the mean age difference also declined after 1990, from 3.5 to 3 years. The trend in England and Wales was similar to that in Canada and the USA (i.e., an upward trend was followed by a downward trend), but the transition to a downward trend occurred later, at around 2000.

Turning to Eastern Europe, we can observe some similarities across the countries in our sample. In Hungary and Poland, for which we have data for the communist period, it is clear that the age difference was steeply decreasing prior to 1990. In all three countries, the smallest age differences were recorded in the early 1990s. Since then, age heterogeneity has again been growing in Eastern Europe, with the mean age difference in Hungary returning to levels recorded in the 1970s. In our group of Western European countries, we can again compare only France and eastern and western Germany. It is, however, noteworthy that while the age difference has declined in western Germany since the early 1990s, from around 3.6 years to 3.2 years; the trend in eastern Germany has followed the pattern in Eastern Europe, with the mean age difference increasing since 2000.

Our findings for parental age differences are much less systematic by country group than the outcomes for quantum differences. Except in Eastern Europe, we can observe a general tendency in recent years toward a decline in mean parental age differences. Yet it does not seem possible to explain the current parental age differences simply by citing differences in gender equality levels. A case in point is Japan, which currently has the smallest parental age differences, despite having rather low levels of gender equality.

While we observe quite diverse patterns in age differences across countries and country groups, the underlying demographic mechanism is in all cases gender differentials in postponement. Specifically, in all of the countries we study, the mean age at childbirth has been increasing continuously since 1990 for both men and women (see supplementary materials for details); i.e., both men and women have been postponing childbirth. In combination with a higher mean age at childbirth for men, this finding suggests that changes in the parental age gap at childbirth are due to differentials in the speed at which postponement advances: if it progresses more quickly among men than among women, the age gap increases; but if, on the other hand, it advances more rapidly among women than men, the age gap decreases. For instance, in Taiwan between 2000 and 2005, the mean age at childbirth increased for men by around 1 year and for women by 0.6 years; thus, the parental age difference increased, as Fig. 4 shows. Between 2006 and 2010 in Taiwan, postponement slowed down among men, but accelerated among women: i.e., the mean age at childbirth increased by 0.8 years for men and by 1.3 years for women, which led to a decline in the average age difference.

## Conclusions and Perspectives

Based on a new, extensive database on male fertility in 17 high-income countries, we explore trends in the differences in the quantum and timing of male and female fertility. We find that, first, the level of male fertility relative to the level of female fertility can vary considerably across countries and over time. Male fertility, as measured by the total fertility rate (TFR), can be both higher and lower than female fertility; although in recent years it has generally been lower rather than higher. This observation is consistent with the results of Schoumaker (2019) based on survey and census data, and our further analysis for a subset of six countries shows that it also holds for cohort fertility rates (CFRs).

Our counterfactual calculations provide evidence for the claim that temporal fluctuations in the ratio between male and female TFR are often driven by age differences between partners and differences in postponement behavior, and our analysis of CFRs also supports this conclusion. Our second main finding is that age differences between fathers and mothers stayed constant or decreased in most of the countries in our study, except in the Eastern European countries and eastern Germany. While this general tendency is in line with expectations based on gender theories, the differences that currently exist across countries seem to be affected by more factors than just differences in gender equality levels.

While our findings indicate that male fertility has generally been lower than female fertility in recent years, this trend does not seem to hold globally. Schoumaker (2017, 2019) showed that in contexts in which polygyny is practiced and populations are rapidly growing, male fertility levels can sometimes be twice as high as female fertility levels. His results suggest that this pattern is driven to a large extent by age differences between couples. This finding is in line with the results of our counterfactual analysis, which indicate that extreme cases tend to disappear when the fathers are assigned to the same cohort as the mothers, and that large gender differentials in fertility levels are often driven by differences in fertility timing. At the other extreme, the lowest TFR ratio reported in the literature is for England and Wales in 1973, at around 0.89 (Schoen 1985). The value we have found for eastern Germany, 0.84, is below this level, and might indicate that eastern German males have been experiencing what Schoen (1985) called a “birth squeeze”: i.e., in eastern Germany, the unequal number of men and women in the reproductive age range is having an impact on the fertility of men. Generally, the variation in TFR ratios we observe across countries and over time is not negligible. Country groups with cultural and/or political similarities seem to have more similar trend patterns. This finding requires further investigation.

The mean age differences between men and women we have detected in our data are close to the orders of magnitude found in other studies (e.g., Kolk 2015). They are, however, lower than the high values observed in some African countries, where mean differences of more than 10 years have been estimated (Schoumaker 2017). Nevertheless, the variation across countries and over time in our 17 high-income countries is substantial. This heterogeneity of age differences is somewhat puzzling. In particular, our findings suggest that while European countries that score high on the gender equality index have smaller age differences, Japan does not seem to fit this picture. Even though Japan scores low on gender inequality indices (World Economic Forum 2017), the age differences among parents in this country are the lowest we have found in our study. This finding requires further investigation. Another factor that might be important is the cross-country variation in the share of migrants and the parental age differences that prevail in their countries of origin. We are, however, unable to study this potential factor, given that for most of the countries and years in our sample, we are unable to separate births by nationality or migration background status. We thus leave the exploration of this question to future research.

Looking at gender differences in fertility postponement, we find that gender differences tend to decrease over time, except in Eastern Europe, where age differences have been increasing. In Eastern Europe, this process has been accompanied by a process of societal restoration (Fodor and Balogh 2010). We also find that in some countries (e.g., Finland and France), the mean age difference has been stable over the period for which we have data. Our comparison of countries suggests that the Danish case, described by Nordfalk et al. (2015), is among those with rather large gender differences in fertility postponement. More research is needed to explain this variation across countries.

It is difficult to assess to what degree the observed patterns reflect gender differences in fertility preferences with respect to both the quantum and the timing of fertility. Surveys in low-income countries designed to assess the ideal number of children tend to report that the ideal number is usually much higher than the number of children actually born (Esteve et al. 2020). Longitudinal research has also shown that individuals tend to adapt their family size ideals to the number of children they were able to have (e.g., Kuhnt et al. 2017). This implies that the ideal number of children is not only a reflection of people’s preferences, but is also moderated by triggers and constraints that affect their fertility biographies. For Europe, data from the Eurobarometer survey 2011 indicate that in most countries, men tend to report lower ideal family sizes than women (Testa 2012). These results are generally consistent with our findings, which show that men have lower fertility than women. In addition, our TFR estimates for 2011 are highly correlated across countries with the Eurobarometer results on ideal family size for both men and women (> 0.9). However, the Eurobarometer results also show that in most countries, the gaps between the ideal and the actual number of children are larger for men than for women (Testa 2012). This observation could be interpreted as providing support for the view that men might be less able than women to realize their fertility desires, which could, in turn, be a result of the sex ratio imbalances.

Regarding preferences on the timing of births, the Eurobarometer survey data show that across member states of the European Union (EU25), both the ideal age to become parent and the latest age at which a person should have children are higher for men than for women (Testa 2006). The ideal age to become a parent is on average around 2 years higher for men than it is for women (Testa 2006), with women setting the ideal age for men higher (about 27.5 years) than men themselves (around 27 years). For the latest age at which a person should have children, the gaps between women and men are even larger. Both women and men set the age deadline for women on average at around 41 years, while both men and women set the age deadline for men at around 46 years (Testa 2006). Billari et al. (2011) used data from the European Social Survey and found slightly higher mean numbers both for women (41.7 years) and men (47.3 years), with some variance across countries. These survey results are generally in line with the gaps we identified. They are also in line with our finding that fertility decreases for men as well after age 45, even though many men are biologically able to have children at ages above 45.

The main outcomes we study in this paper—the TFR and the mean age at childbirth—are not the only measures of the quantum and the tempo of fertility. For instance, the proportion of childless individuals and the age at first birth have also received considerable interest in the literature. While this research has mostly focused on women, a small number of studies have also included men (Paavilainen et al. 2016). However, one of the limitations of the register data we employ is that we cannot use them to calculate the number of childless individuals. In addition, analyses by parity, such as by first birth, are only possible for women, if at all, as none of the birth registers records the parity of the father. Moreover, the mean parental age difference is not the only measure of the extent to which the paternal and the maternal age differ (e.g., Kolk 2015). However, using other measures, such as the variance or the standard deviation of the age difference, requires information on the joint age distribution of mothers and fathers, which cannot be derived from the ASFRs we provide as part of the HFC.

The new database we created to study male fertility is not limited to the questions we have investigated in this paper. It can, for example, be used in macro-level investigations of associations between male fertility levels and important economic and social indicators, including gender equality measures. There are many influential publications that have studied these relationships among women (e.g., Brewster and Rindfuss 2000; Myrskylä et al. 2009), and comparing the outcomes for female fertility with the results for male fertility might provide important insights. Such analyses will likely further improve our understanding of the role of gender in current fertility trends. The database can also be used in comparative analyses of trends in the paternal age at childbirth. Advanced paternal age is an important predictor of health outcomes of children, and has been attracting increasing attention in recent years (e.g., Khandwala et al. 2017). Thus, the database offers many promising avenues for future research, and we invite other researchers to make use of this new data resource available as part of the Human Fertility Collection (2019).

## Electronic supplementary material

Below is the link to the electronic supplementary material.

Supplementary material 1 (PDF 206 kb)
