# Supplementary material for: Male–Female Fertility Differentials Across 17 High-Income Countries: Insights From A New Data Resource
Source: Eur J Popul. 2021 Jan 20;37(2):417–41. doi: 10.1007/s10680-020-09575-9 (PMC8035372; doi:10.1007/s10680-020-09575-9)
Supplement: Supplementary file 1 — Supplementary material 1 (PDF 206 kb) [file 10680_2020_9575_MOESM1_ESM.pdf]

attachment to manuscript

Male-female fertility differentials across 17 high-income countries: Insights from a new data resource based on high-quality birth registers

Supplementary materials

Total fertility rates by country and gender

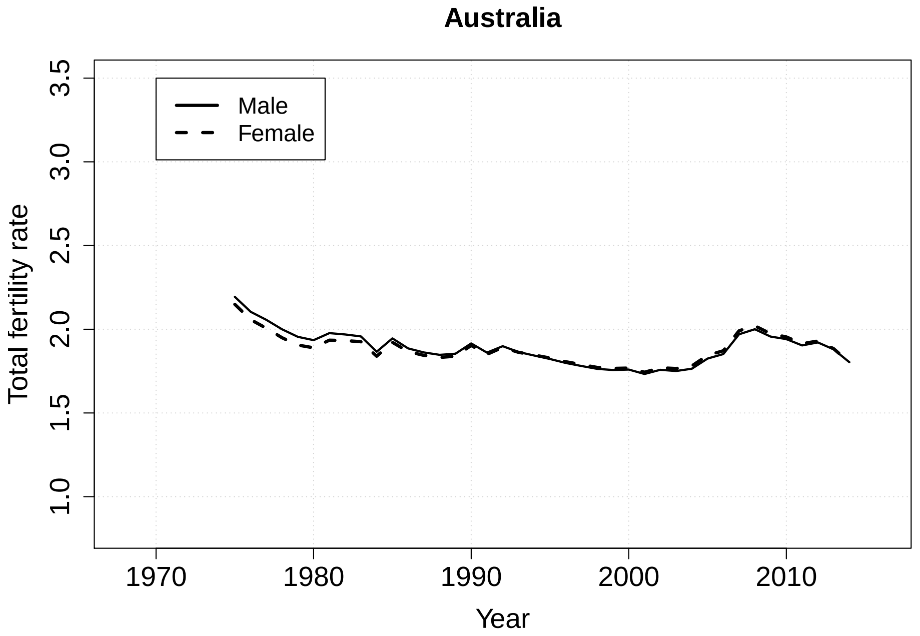

Figure A1: Total fertility rate by gender, Australia.

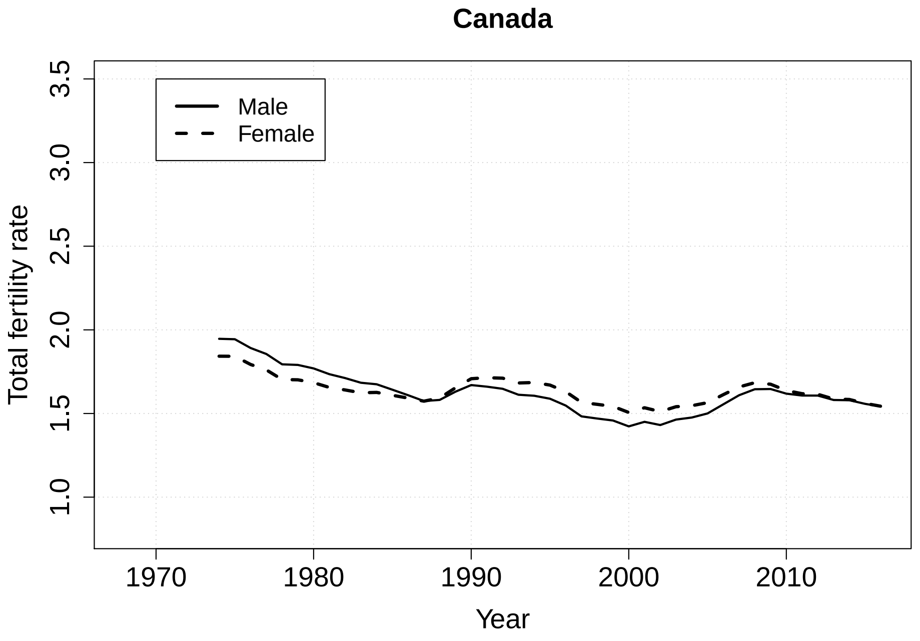

Figure A2: Total fertility rate by gender, Canada.

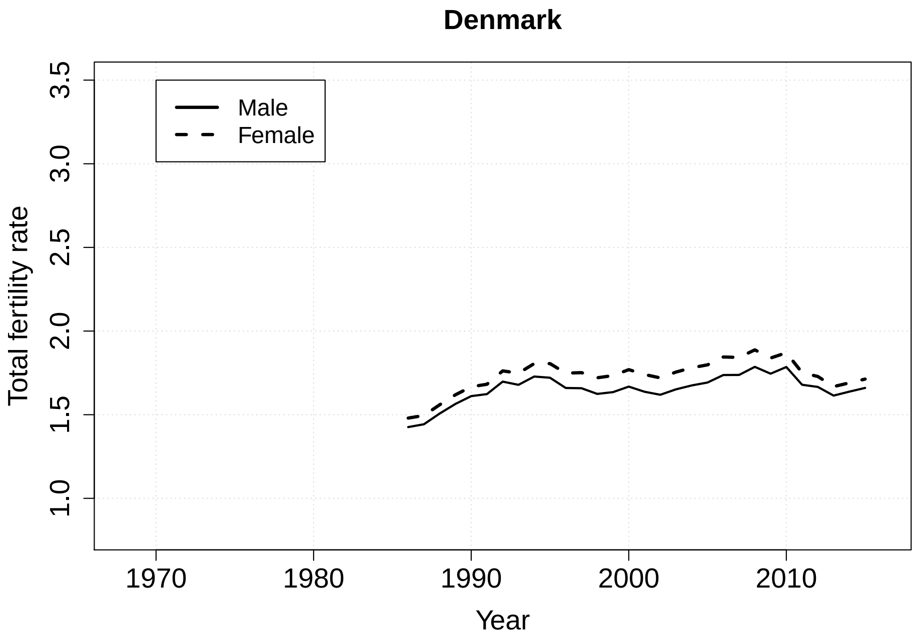

Figure A3: Total fertility rate by gender, Denmark.

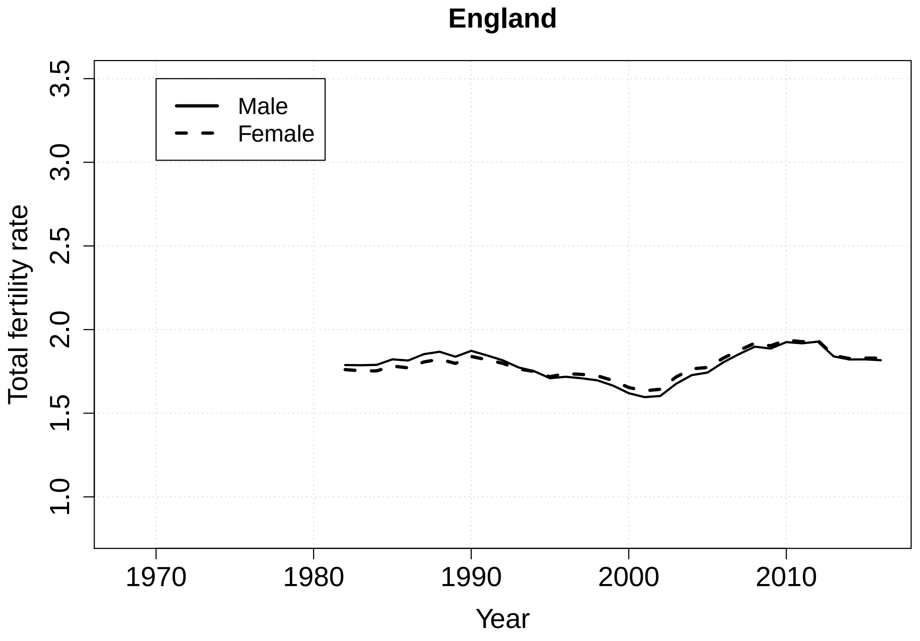

Figure A4: Total fertility rate by gender, England.

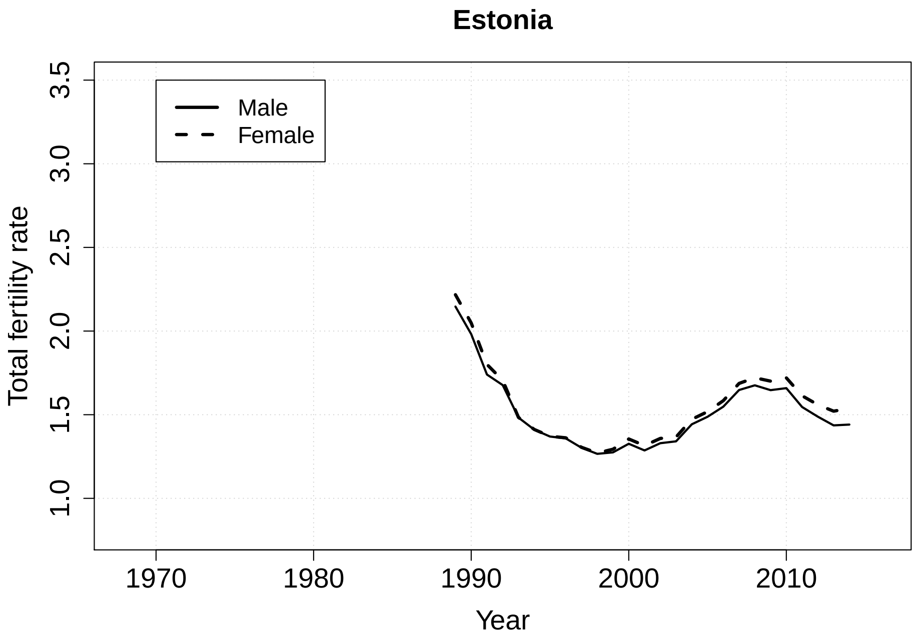

Figure A5: Total fertility rate by gender, Estonia.

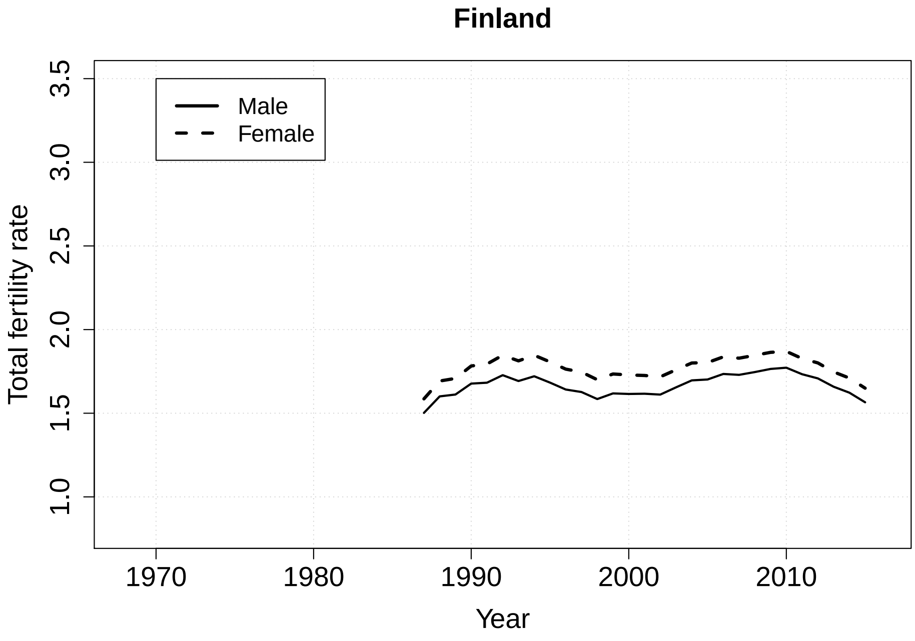

Figure A6: Total fertility rate by gender, Finland.

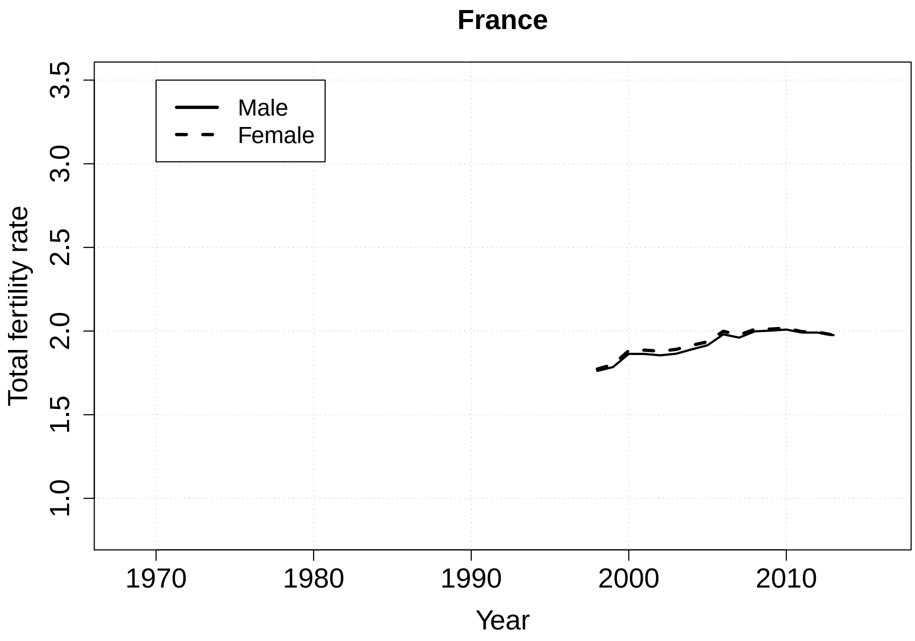

Figure A7: Total fertility rate by gender, France.

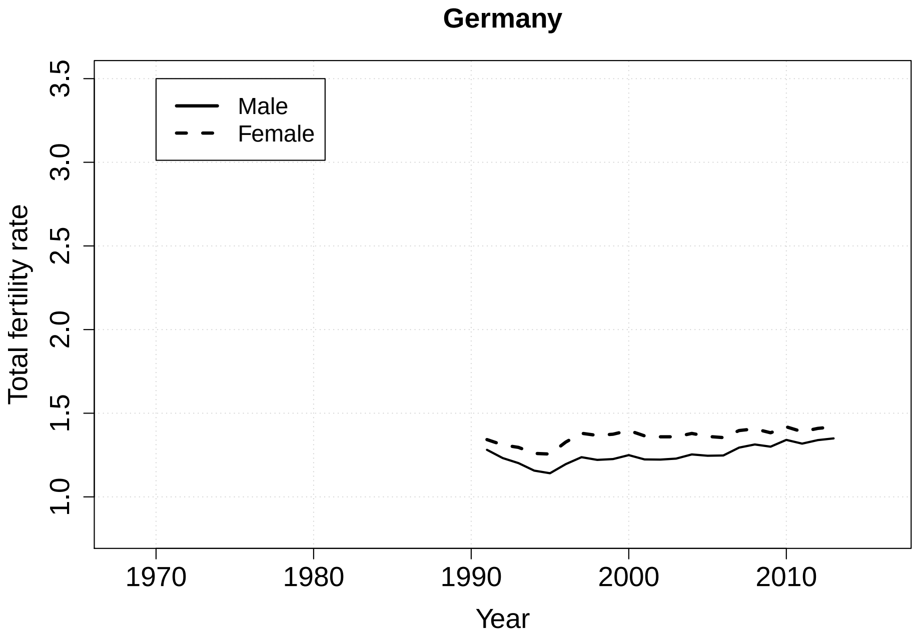

Figure A8: Total fertility rate by gender, Germany.

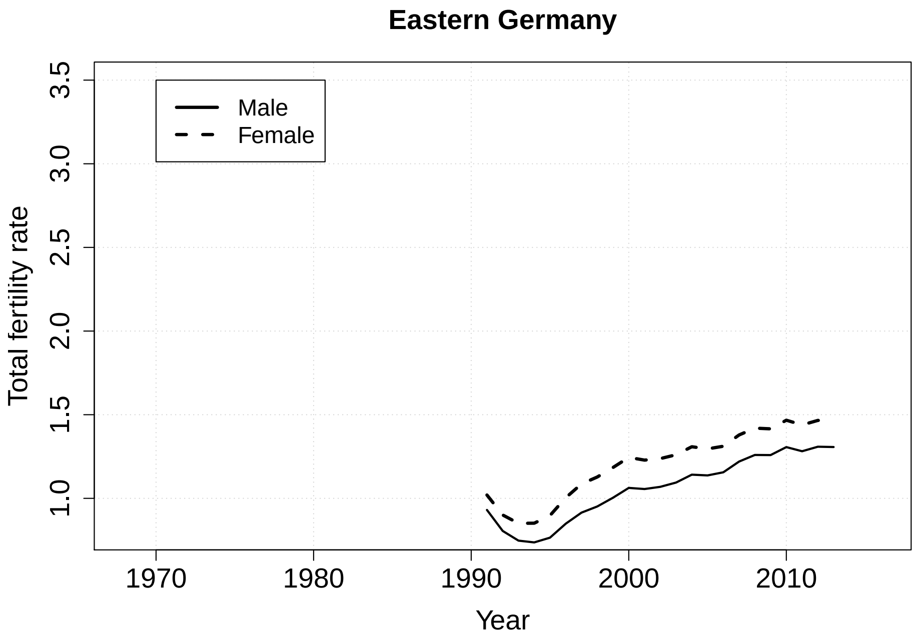

Figure A9: Total fertility rate by gender, eastern Germany.

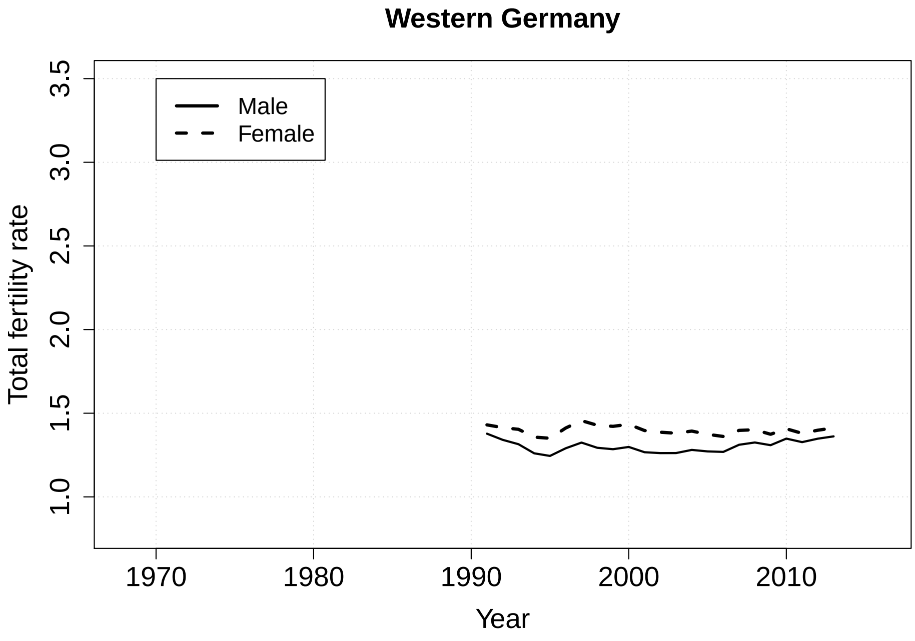

Figure A10: Total fertility rate by gender, western Germany.

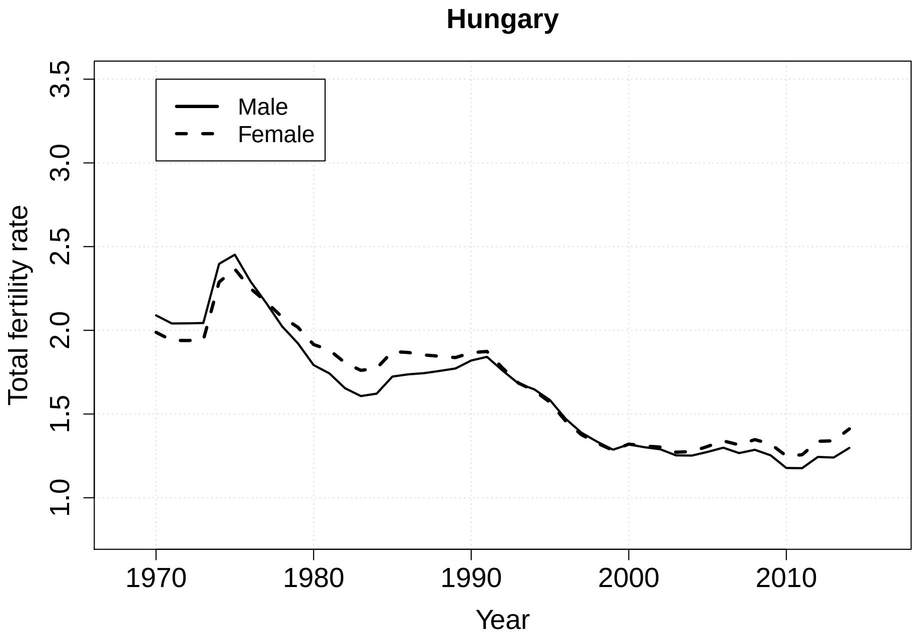

Figure A11: Total fertility rate by gender, Hungary.

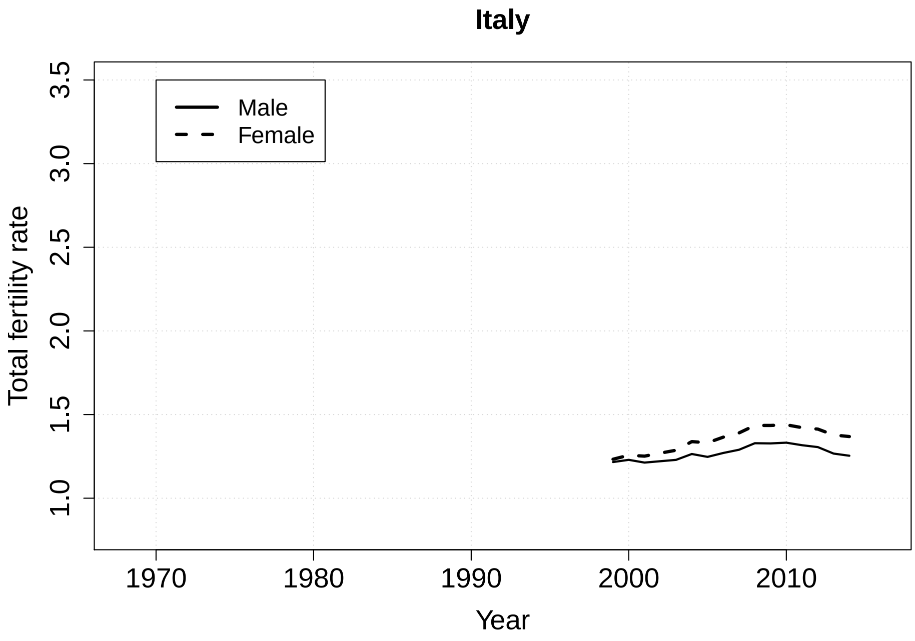

Figure A12: Total fertility rate by gender, Italy.

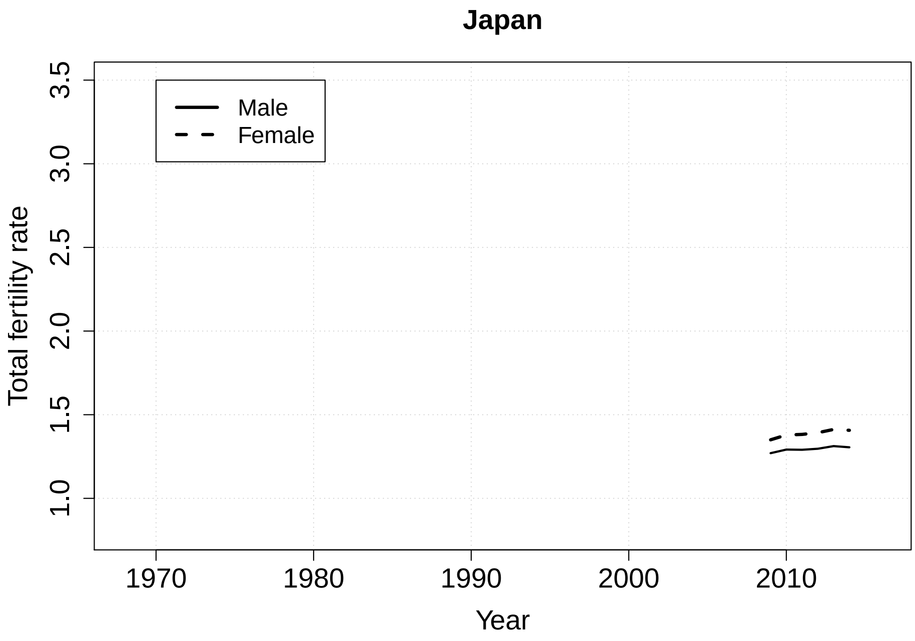

Figure A13: Total fertility rate by gender, Japan.

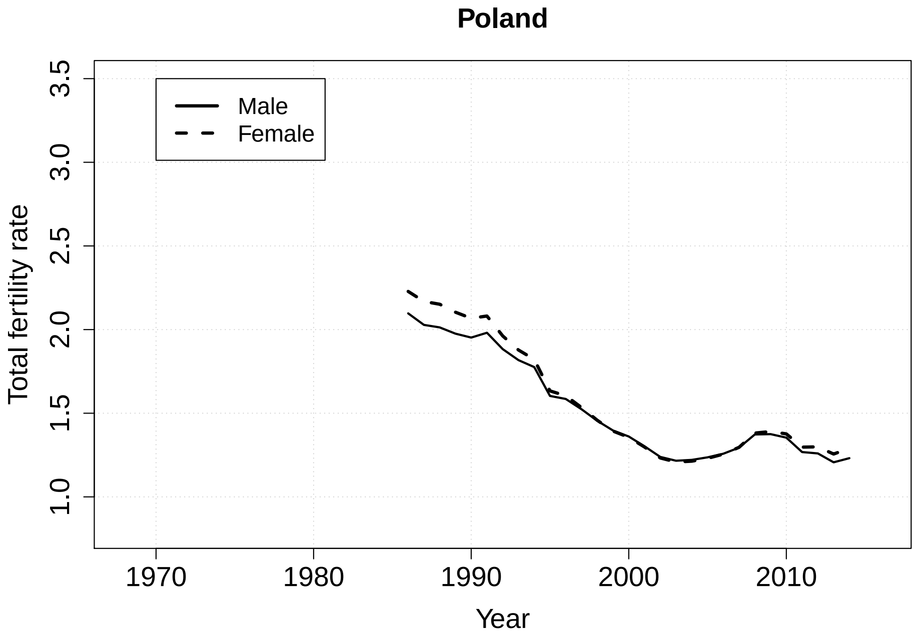

Figure A14: Total fertility rate by gender, Poland.

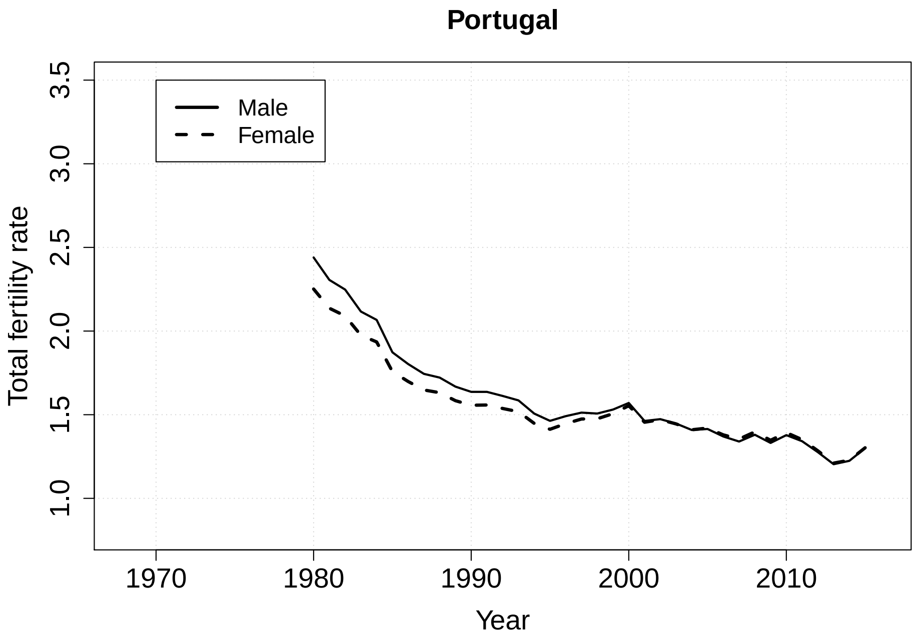

Figure A15: Total fertility rate by gender, Portugal.

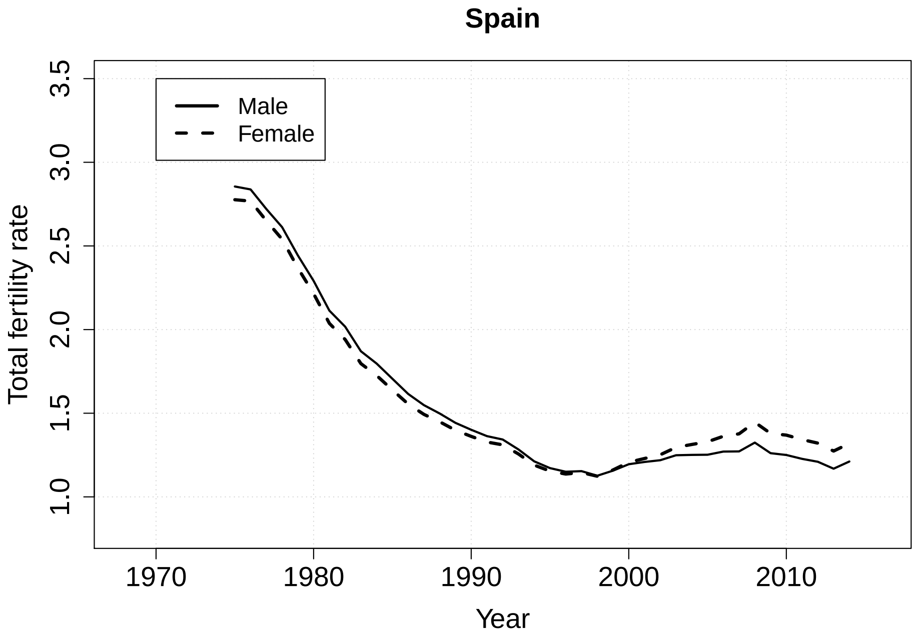

Figure A16: Total fertility rate by gender, Spain.

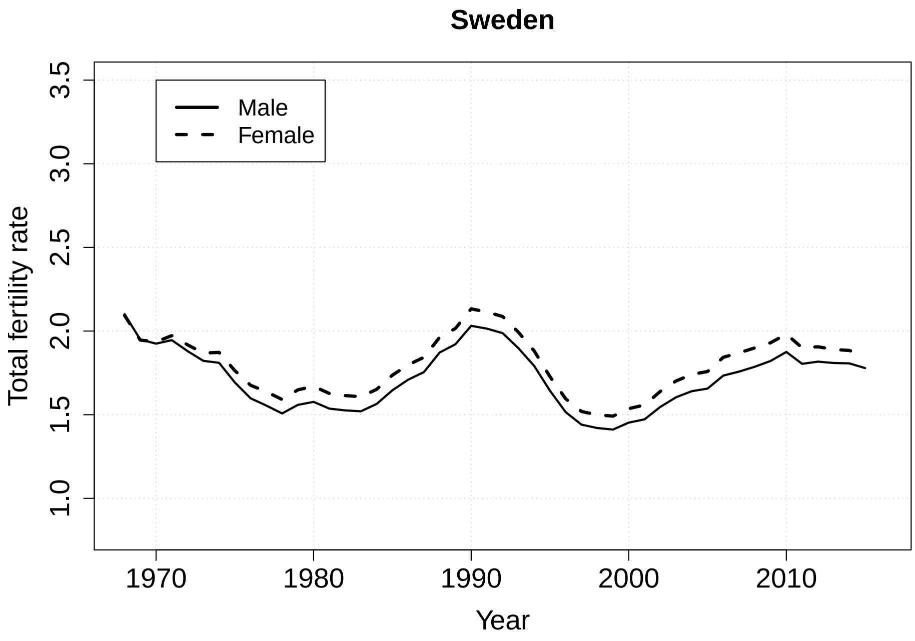

Figure A17: Total fertility rate by gender, Sweden.

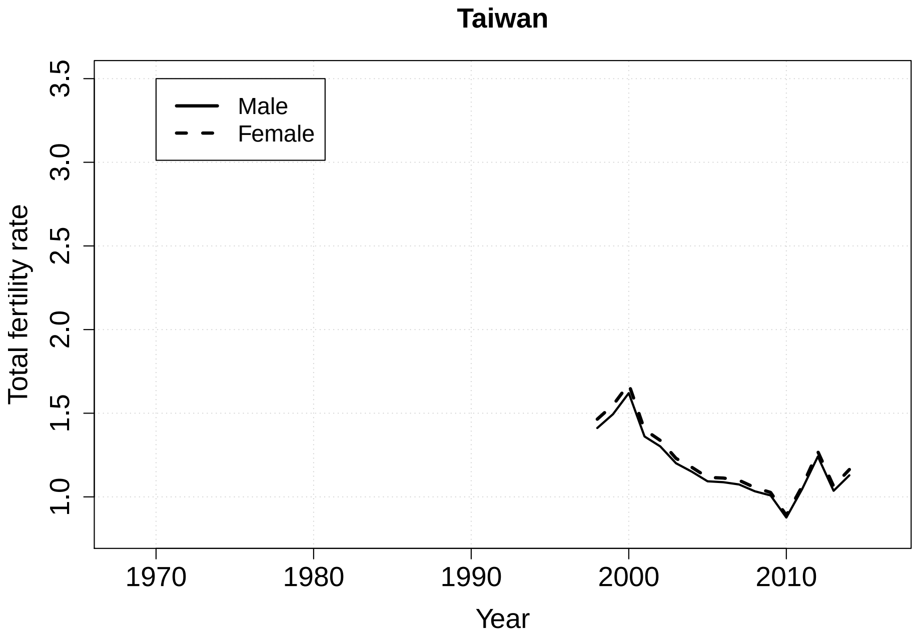

Figure A18: Total fertility rate by gender, Taiwan.

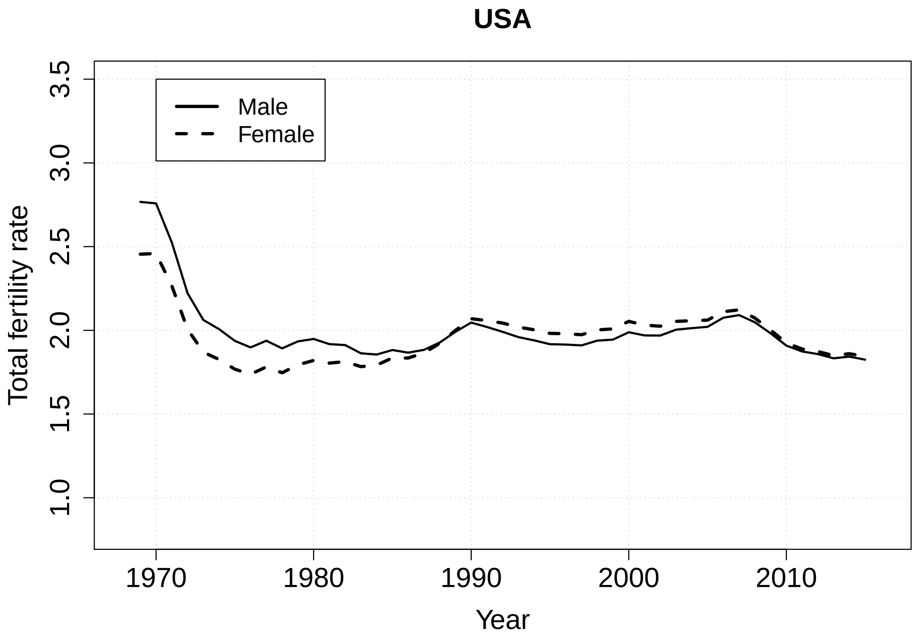

Figure A19: Total fertility rate by gender, USA.

Hypothetical male TFR/female TFR ratios

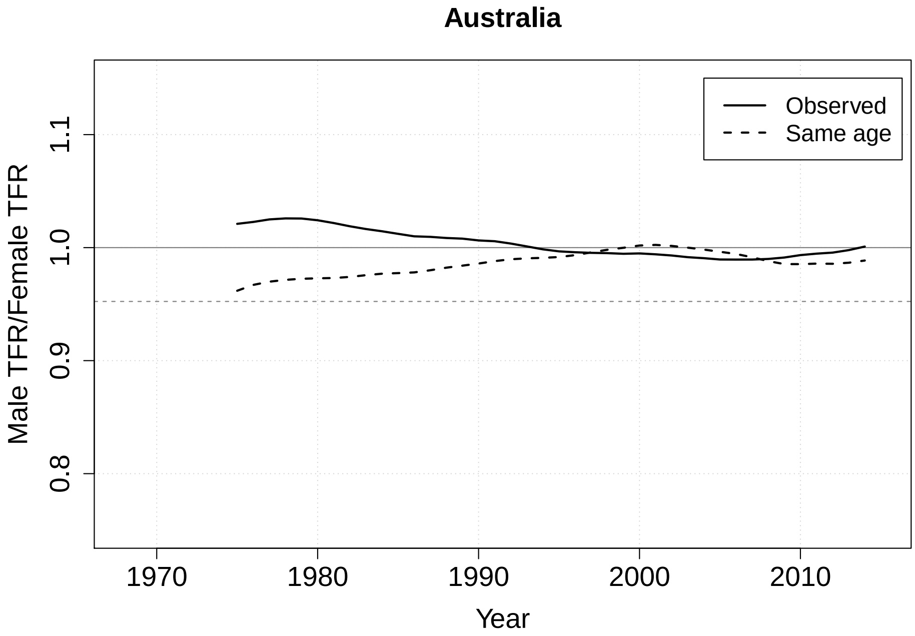

Figure B1: Hypothetical Male/Female TFR ratios in which fathers are assigned the maternal age at birth, Australia.

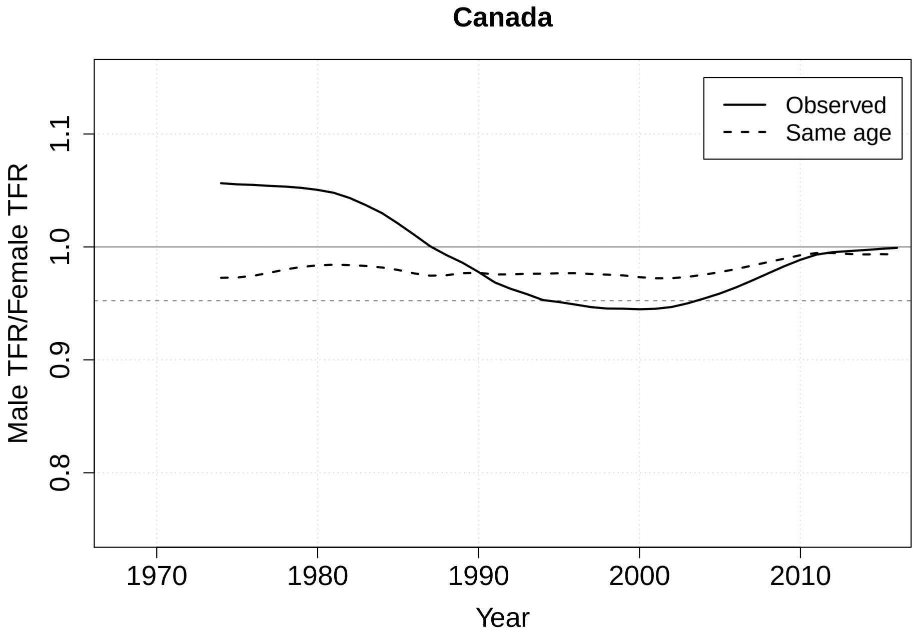

Figure B2: Hypothetical Male/Female TFR ratios in which fathers are assigned the maternal age at birth, Canada.

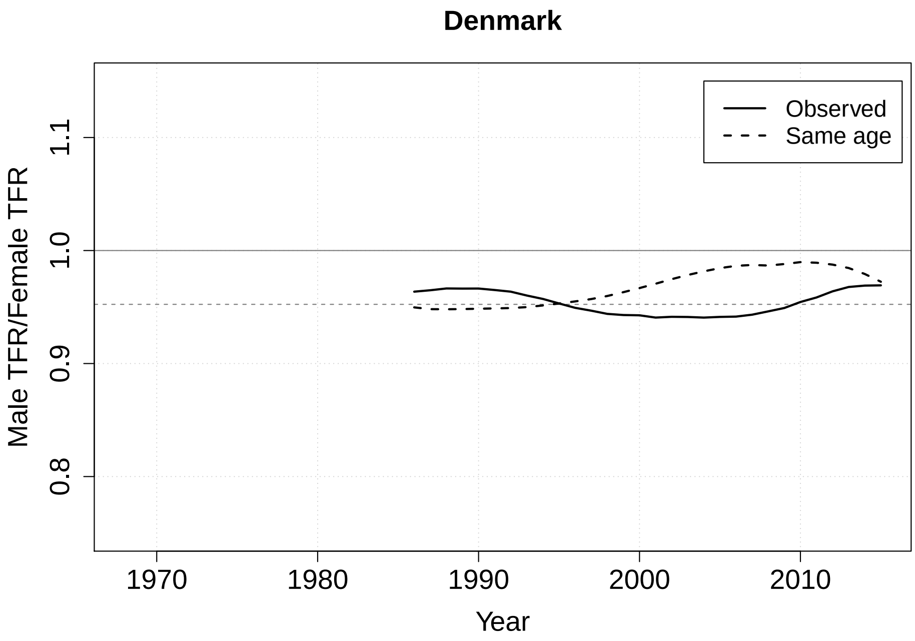

Figure B3: Hypothetical Male/Female TFR ratios in which fathers are assigned the maternal age at birth, Denmark.

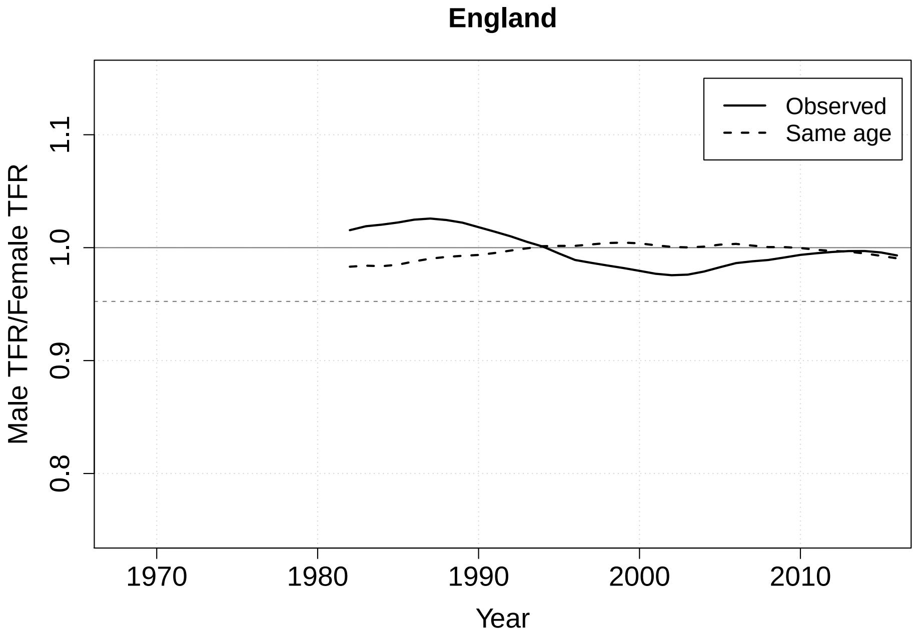

Figure B4: Hypothetical Male/Female TFR ratios in which fathers are assigned the maternal age at birth, England.

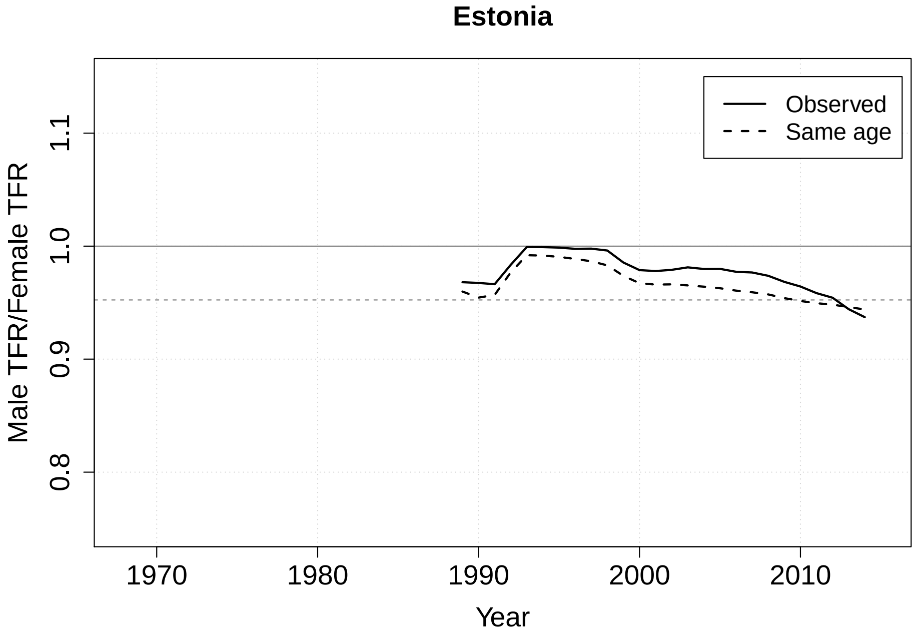

Figure B5: Hypothetical Male/Female TFR ratios in which fathers are assigned the maternal age at birth, Estonia.

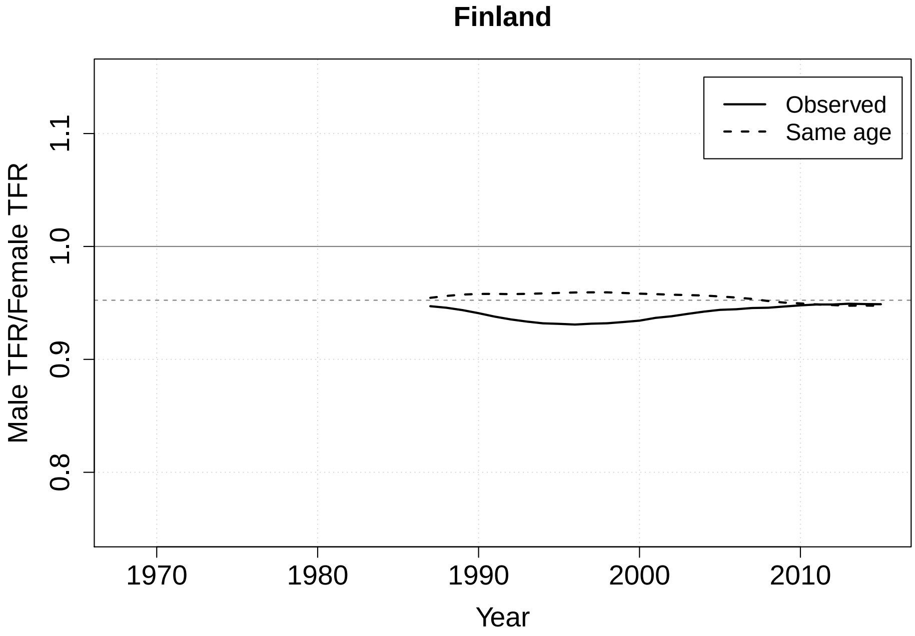

Figure B6: Hypothetical Male/Female TFR ratios in which fathers are assigned the maternal age at birth, Finland.

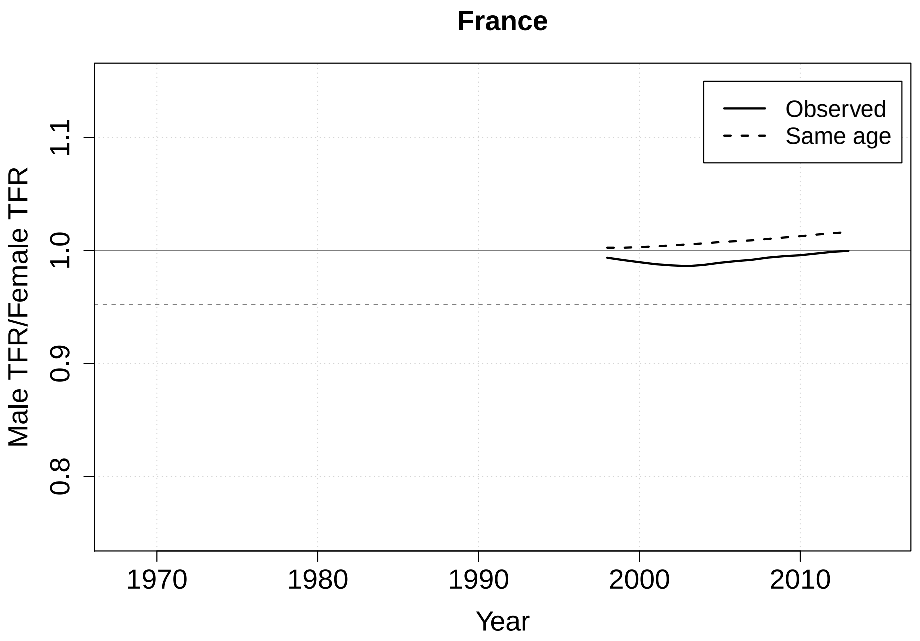

Figure B7: Hypothetical Male/Female TFR ratios in which fathers are assigned the maternal age at birth, France.

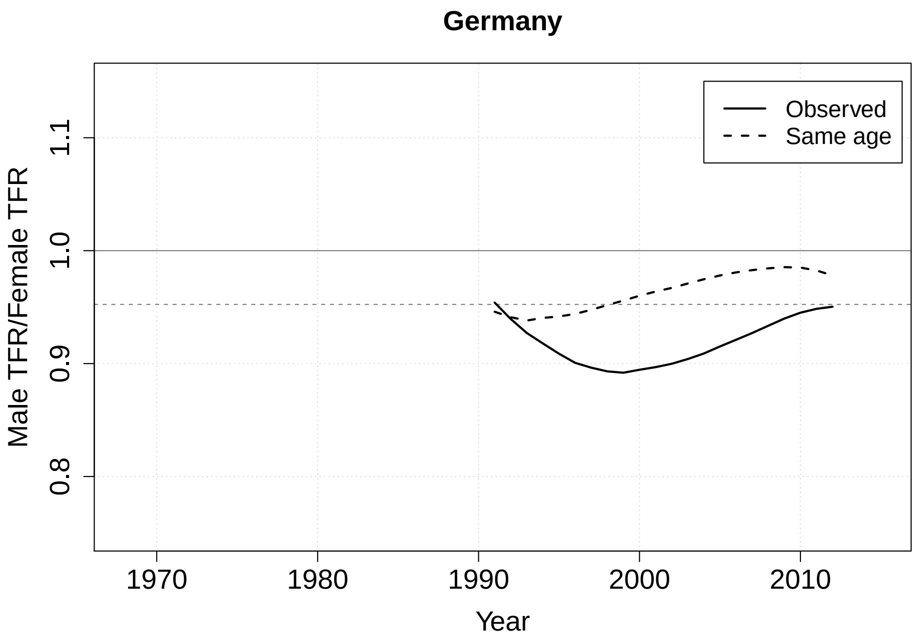

Figure B8: Hypothetical Male/Female TFR ratios in which fathers are assigned the maternal age at birth, Germany.

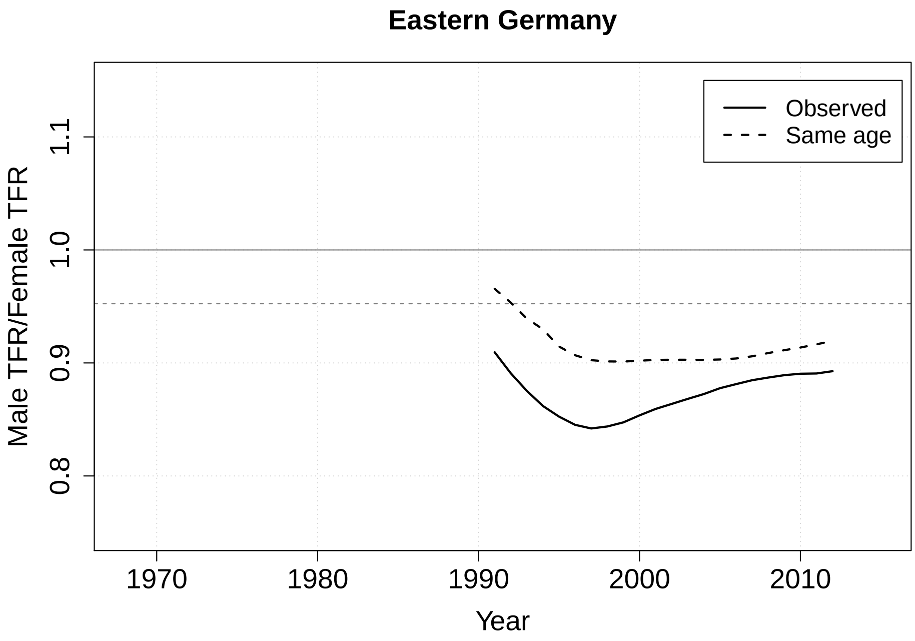

Figure B9: Hypothetical Male/Female TFR ratios in which fathers are assigned the maternal age at birth, eastern Germany.

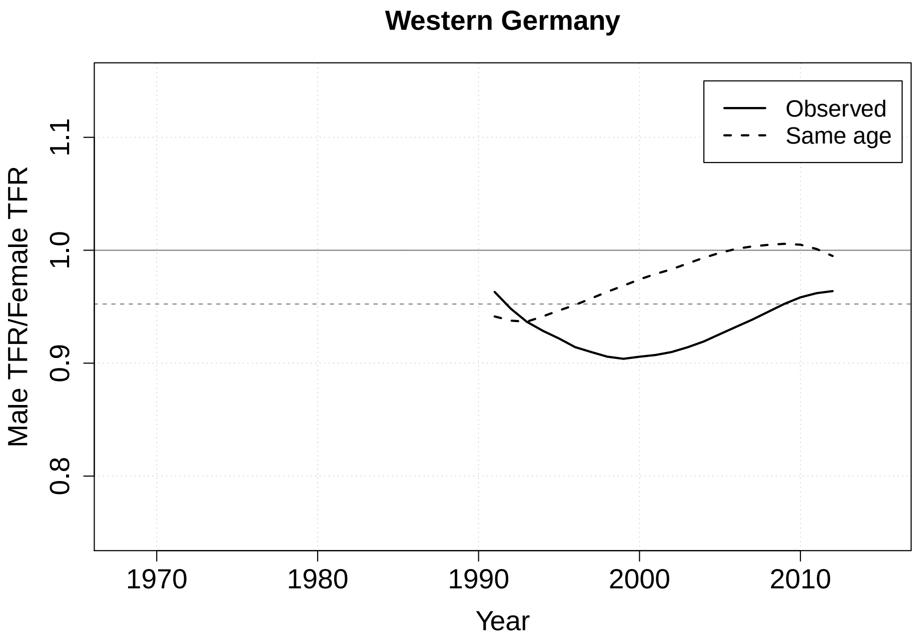

Figure B10: Hypothetical Male/Female TFR ratios in which fathers are assigned the maternal age at birth, western Germany.

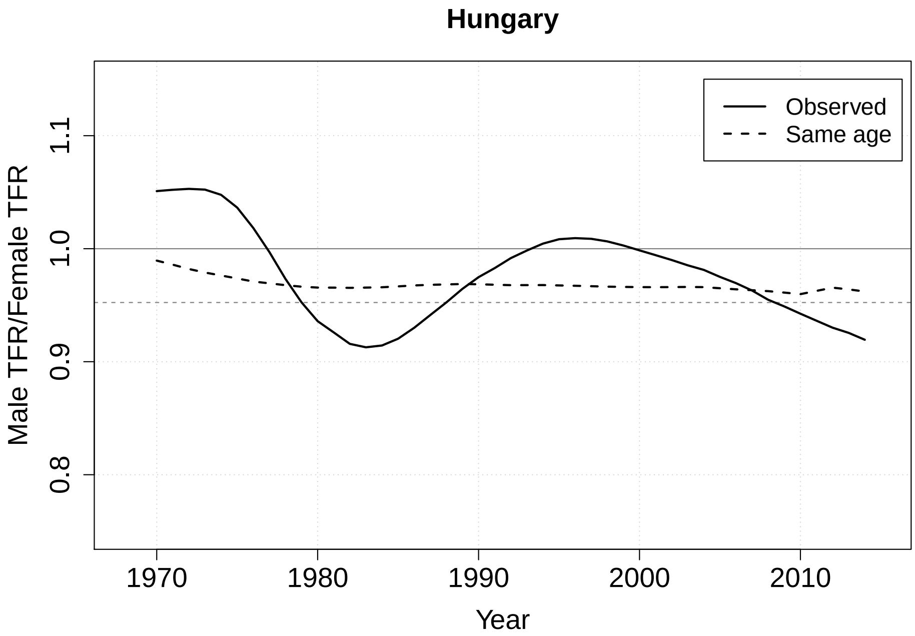

Figure B11: Hypothetical Male/Female TFR ratios in which fathers are assigned the maternal age at birth, Hungary.

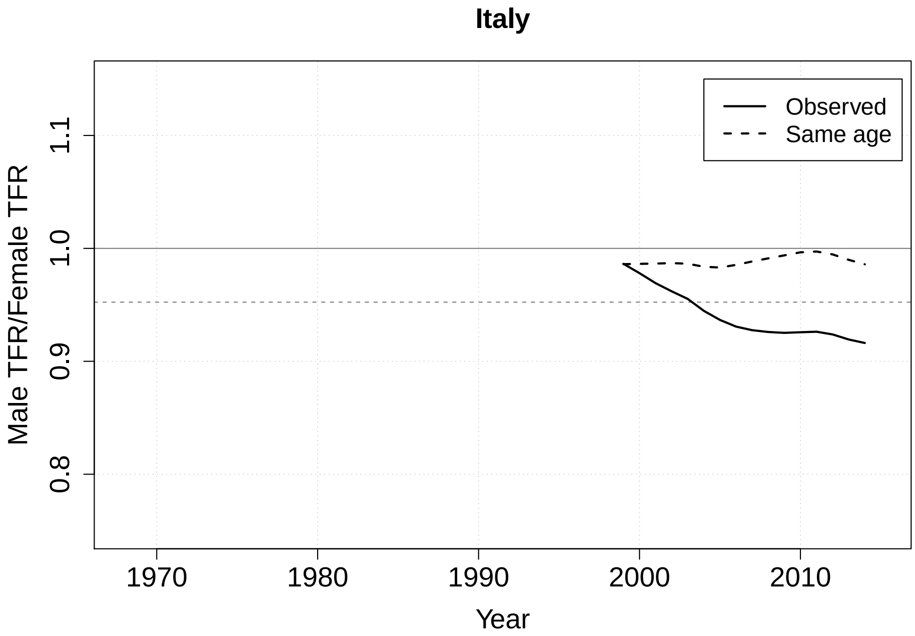

Figure B12: Hypothetical Male/Female TFR ratios in which fathers are assigned the maternal age at birth, Italy.

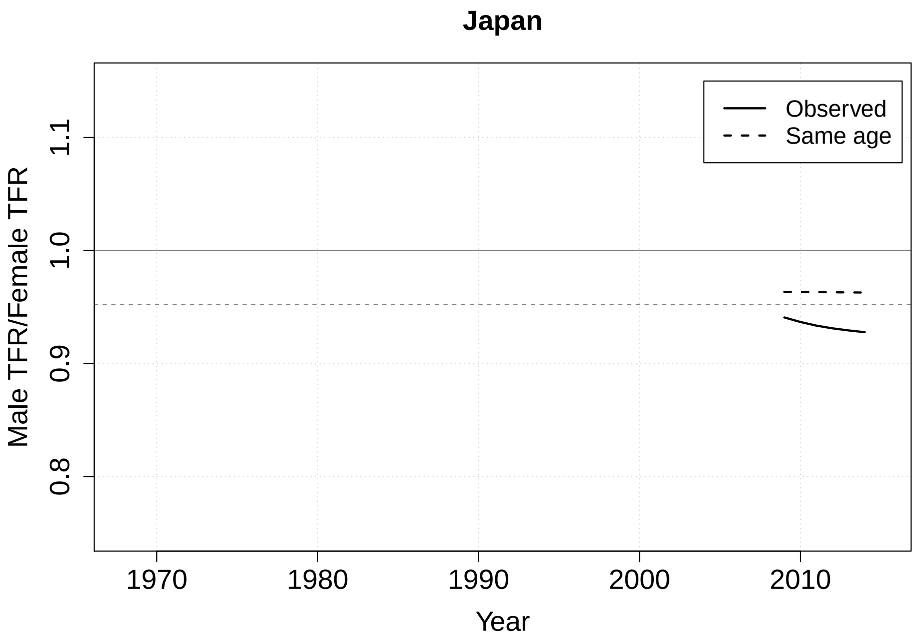

Figure B13: Hypothetical Male/Female TFR ratios in which fathers are assigned the maternal age at birth, Japan.

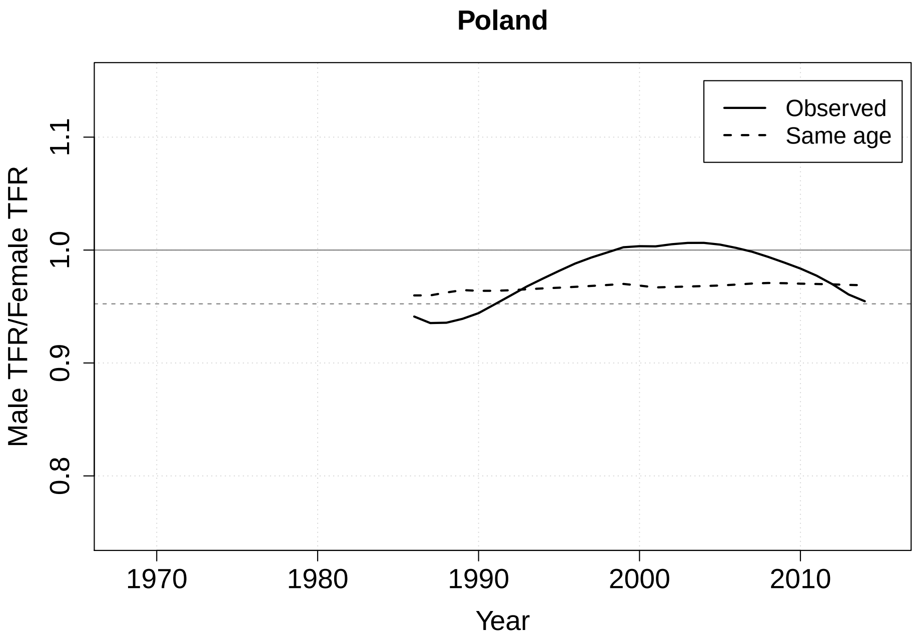

Figure B14: Hypothetical Male/Female TFR ratios in which fathers are assigned the maternal age at birth, Poland.

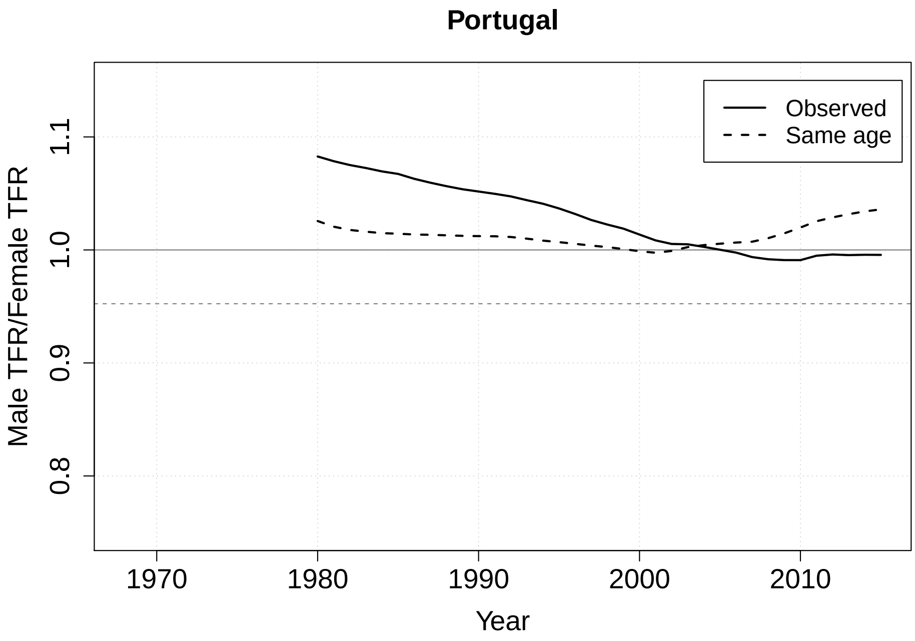

Figure B15: Hypothetical Male/Female TFR ratios in which fathers are assigned the maternal age at birth, Portugal.

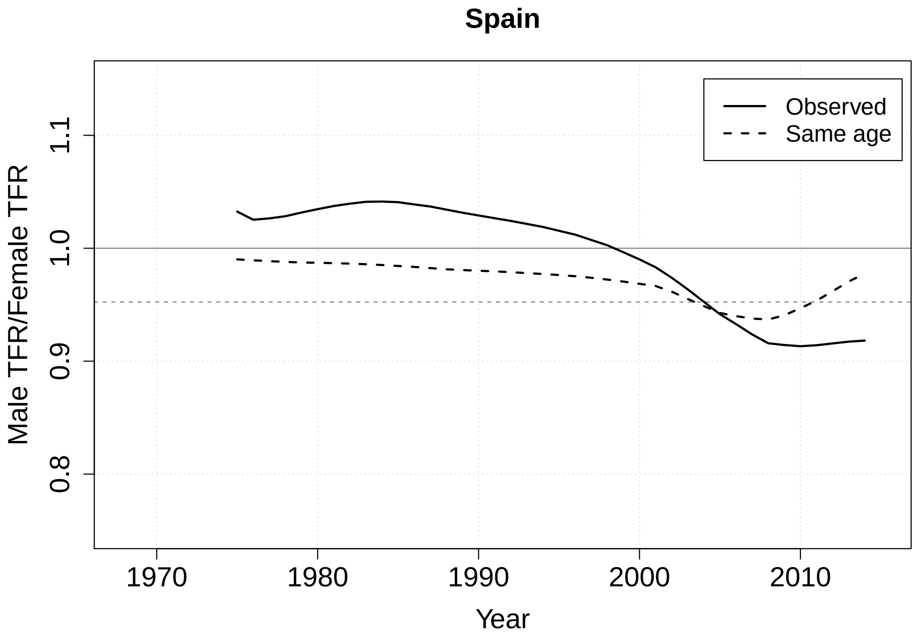

Figure B16: Hypothetical Male/Female TFR ratios in which fathers are assigned the maternal age at birth, Spain.

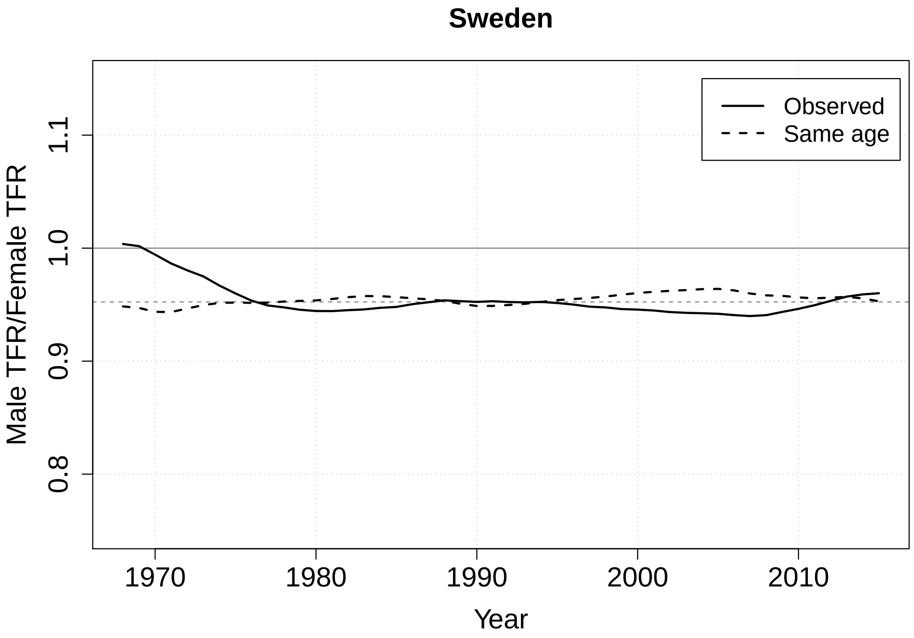

Figure B17: Hypothetical Male/Female TFR ratios in which fathers are assigned the maternal age at birth, Sweden.

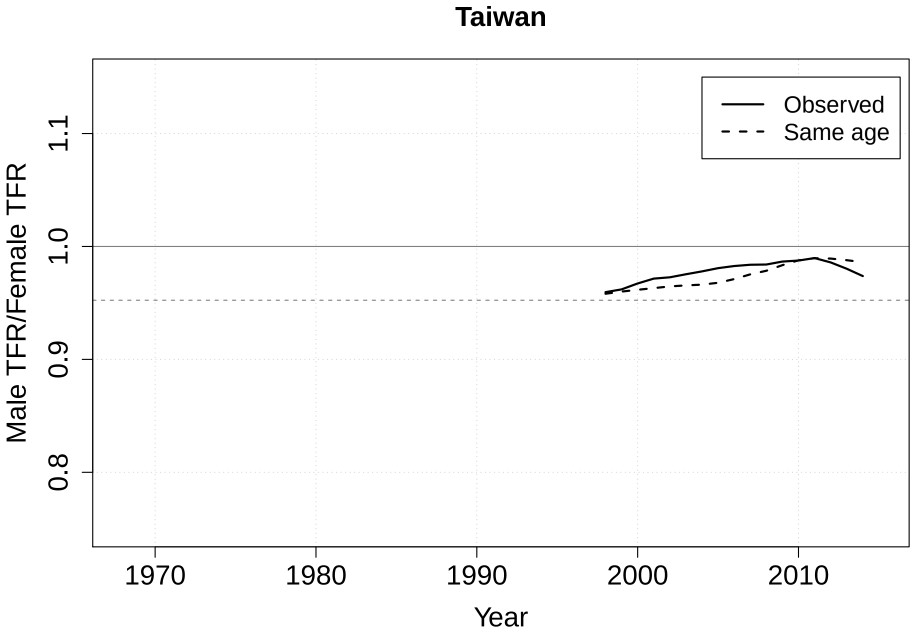

Figure B18: Hypothetical Male/Female TFR ratios in which fathers are assigned the maternal age at birth, Taiwan.

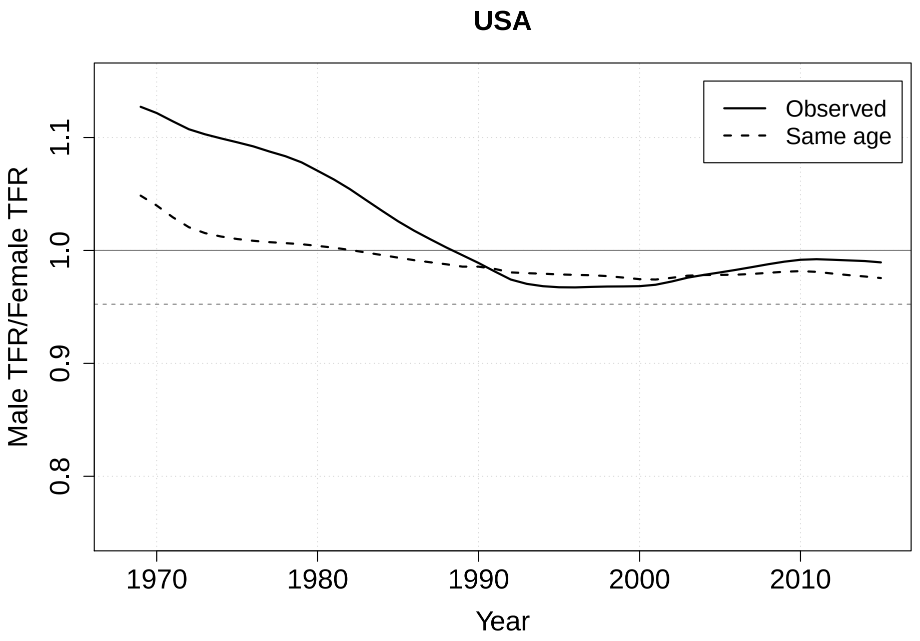

Figure B19: Hypothetical Male/Female TFR ratios in which fathers are assigned the maternal age at birth, USA.

Mean age at childbirth by country and gender

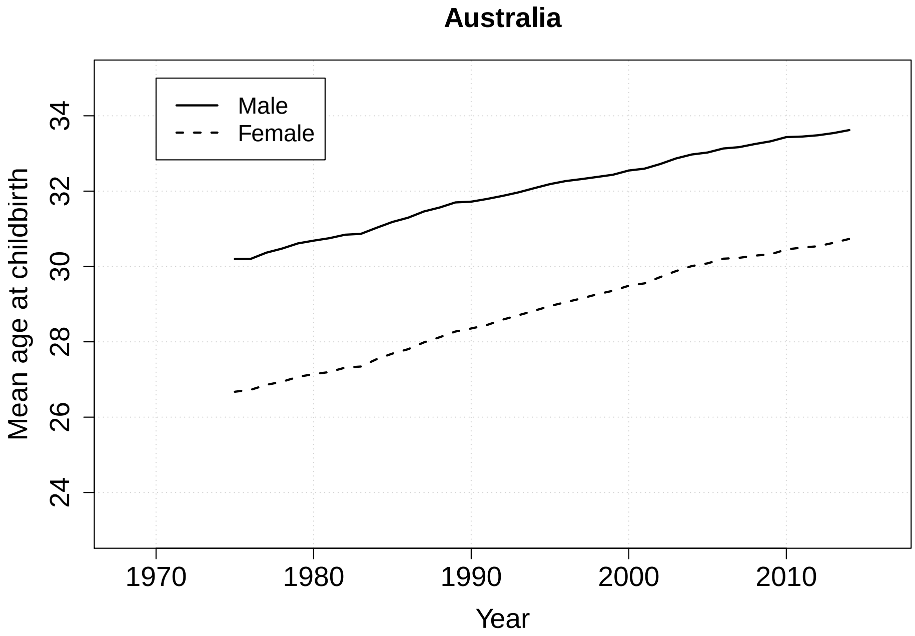

Figure C1: Mean age at childbirth by gender, Australia.

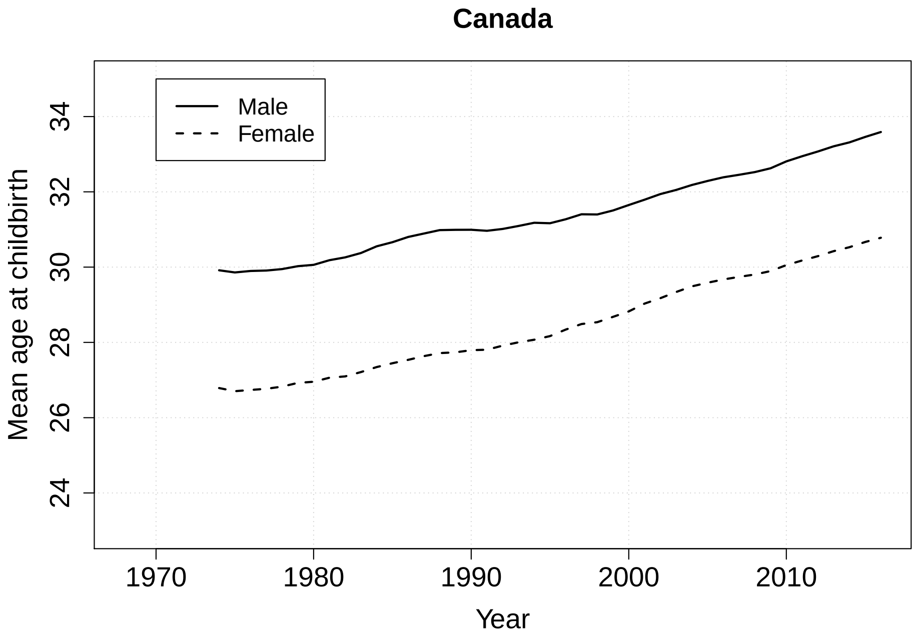

Figure C2: Mean age at childbirth by gender, Canada.

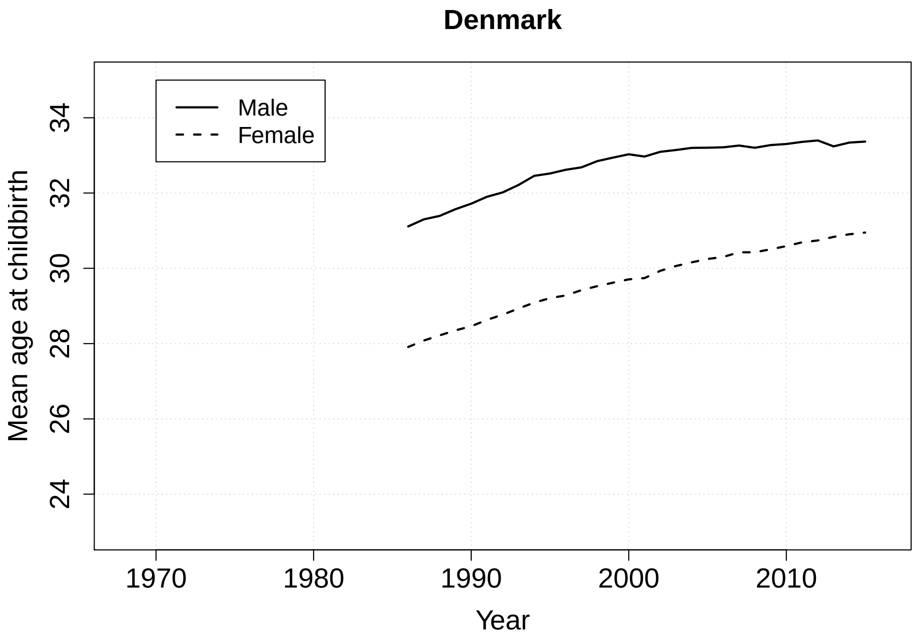

Figure C3: Mean age at childbirth by gender, Denmark.

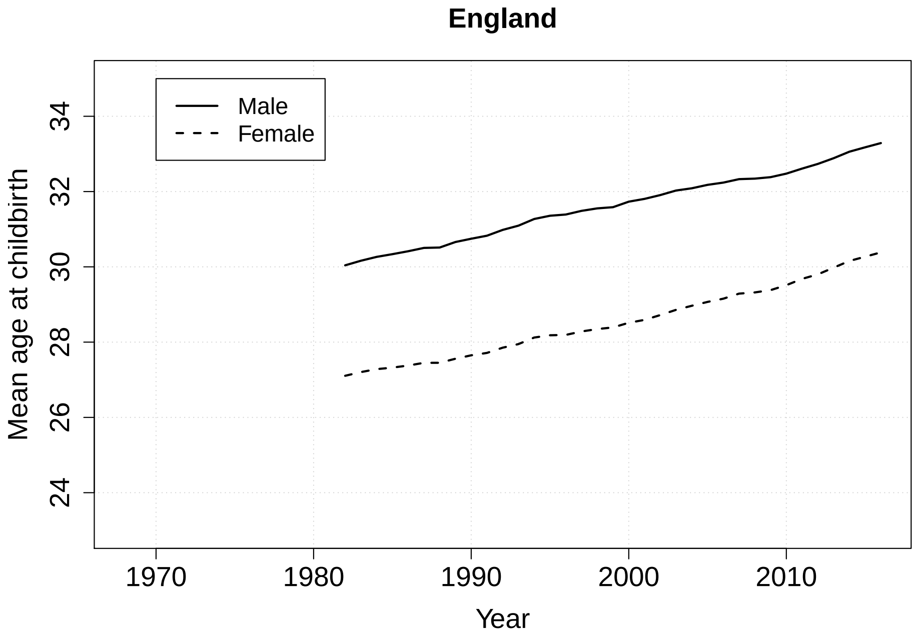

Figure C4: Mean age at childbirth by gender, England.

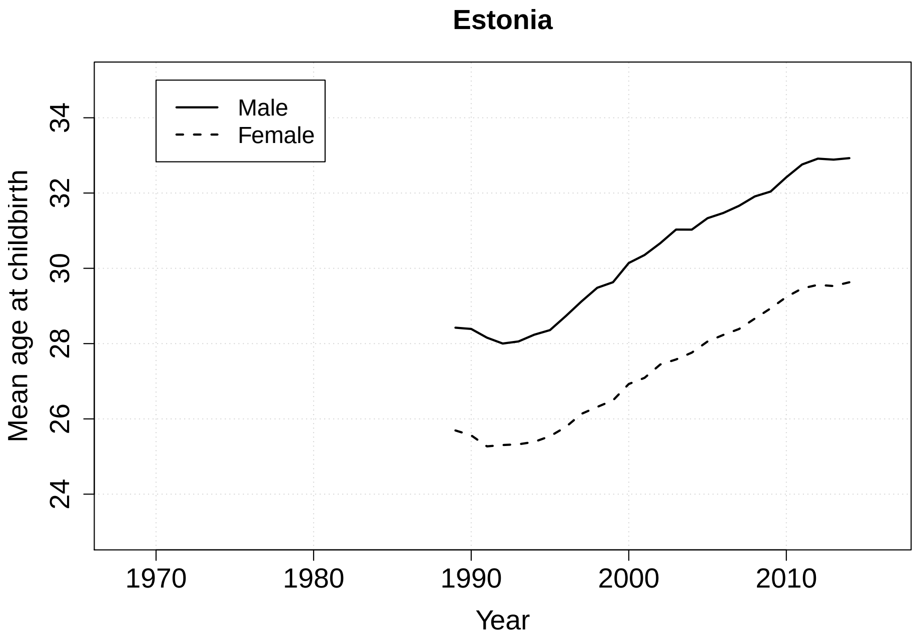

Figure C5: Mean age at childbirth by gender, Estonia.

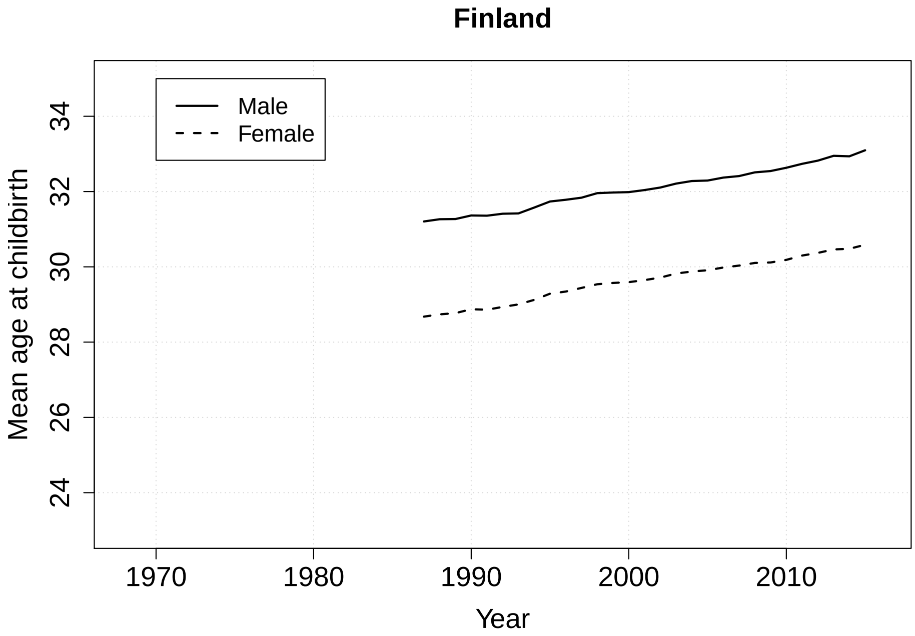

Figure C6: Mean age at childbirth by gender, Finland.

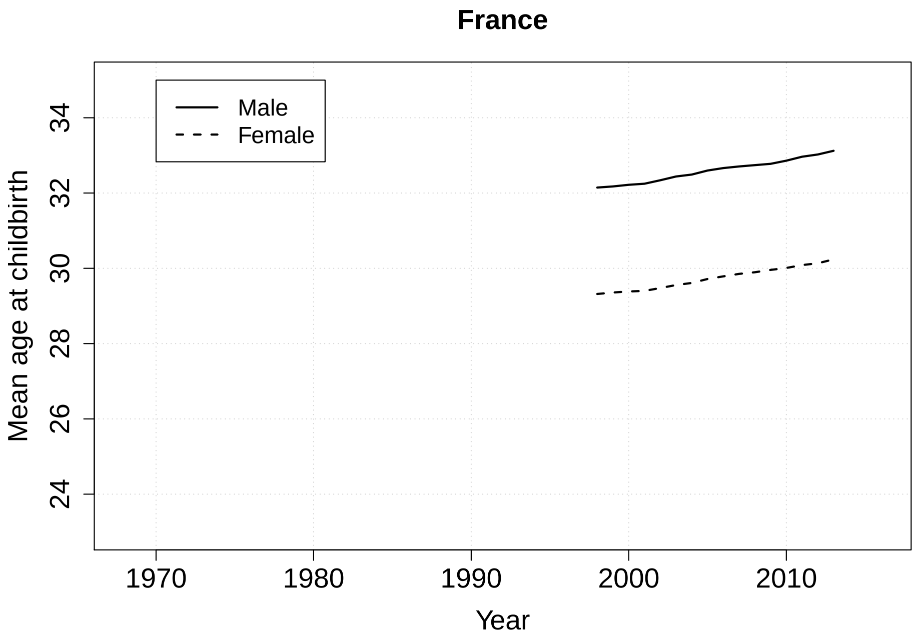

Figure C7: Mean age at childbirth by gender, France.

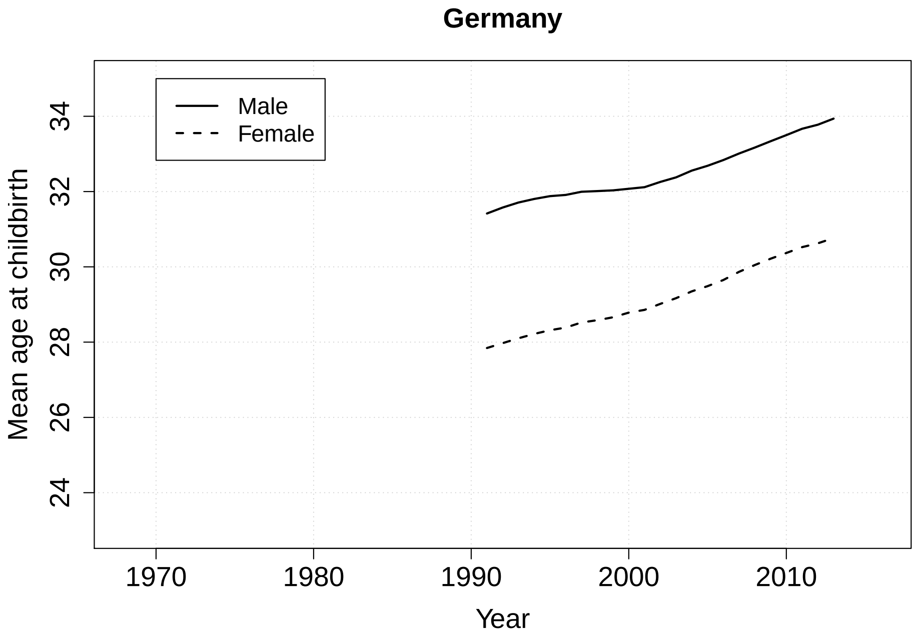

Figure C8: Mean age at childbirth by gender, Germany.

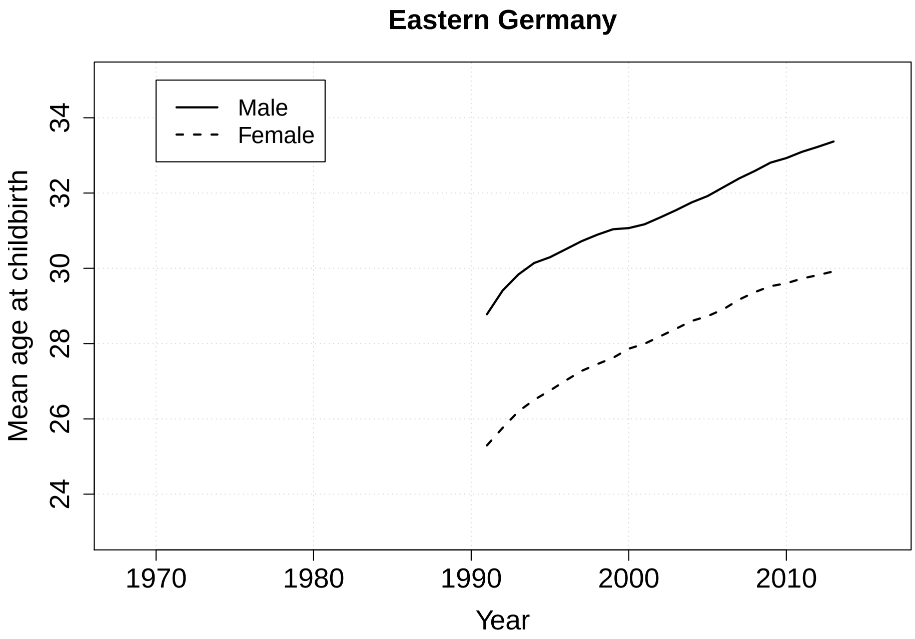

Figure C9: Mean age at childbirth by gender, eastern Germany.

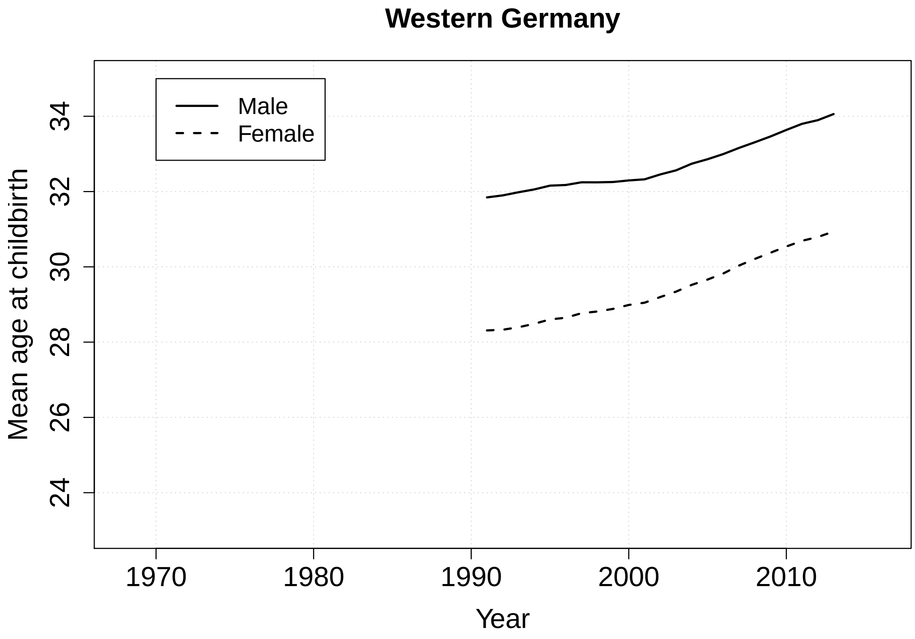

Figure C10: Mean age at childbirth by gender, western Germany.

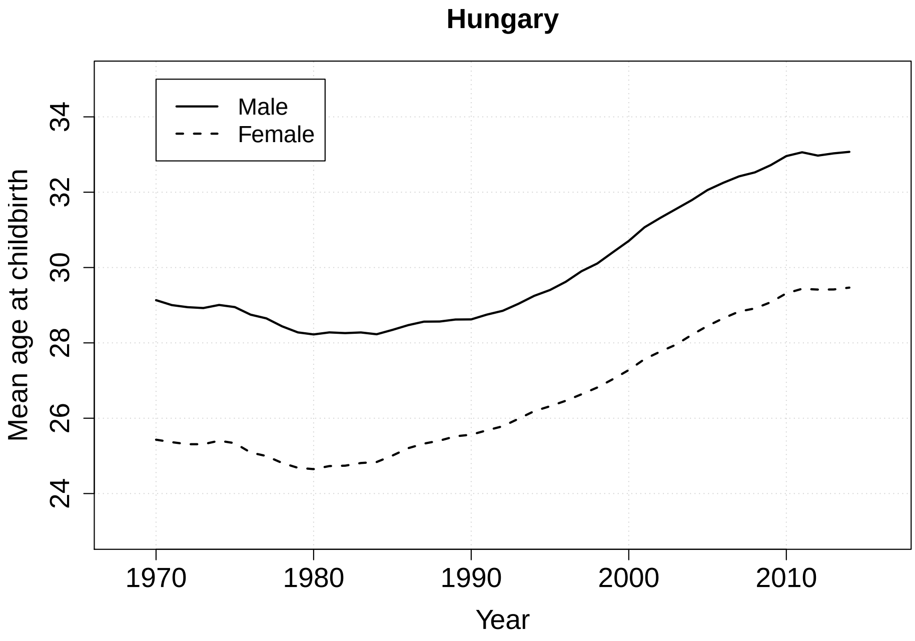

Figure C11: Mean age at childbirth by gender, Hungary.

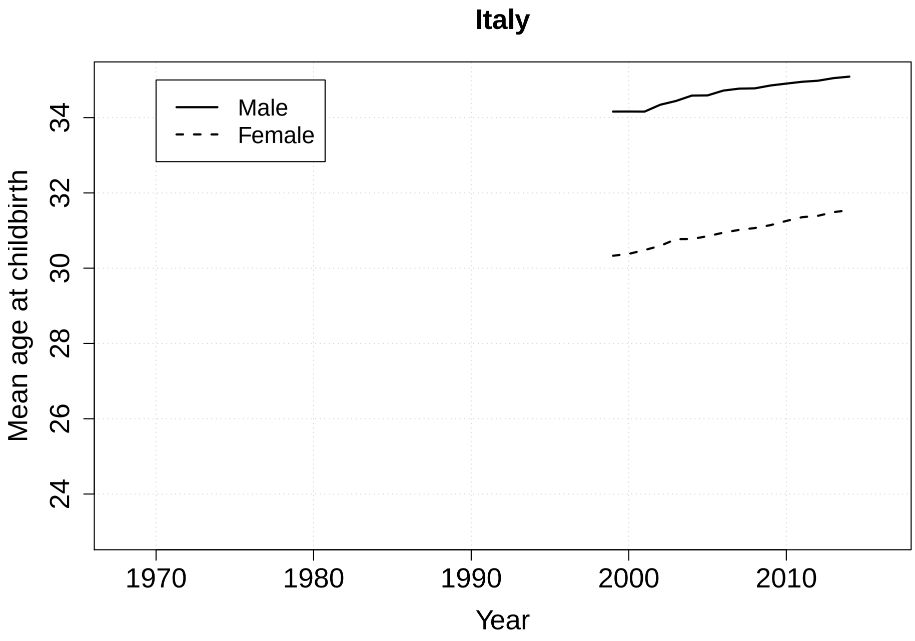

Figure C12: Mean age at childbirth by gender, Italy.

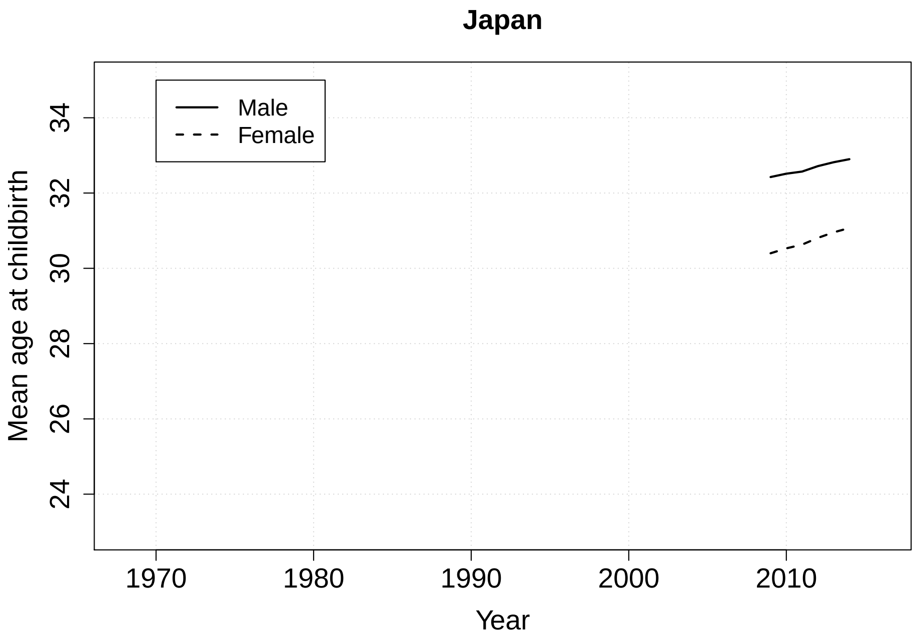

Figure C13: Mean age at childbirth by gender, Japan.

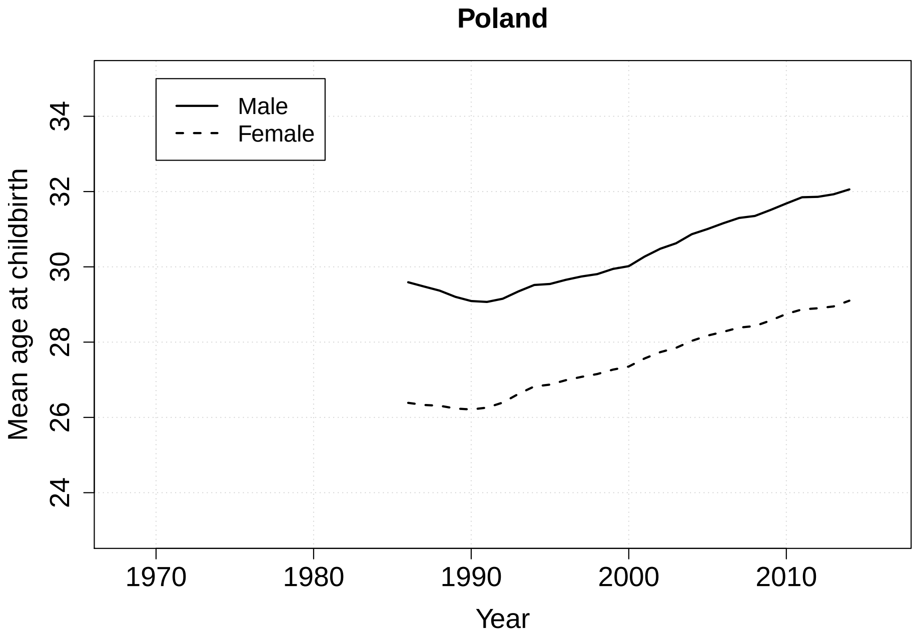

Figure C14: Mean age at childbirth by gender, Poland.

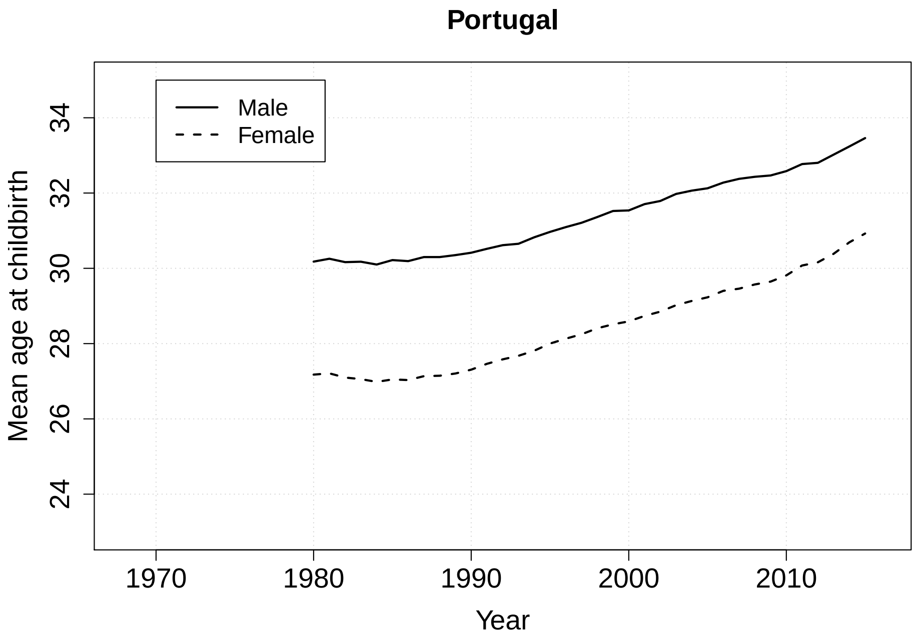

Figure C15: Mean age at childbirth by gender, Portugal.

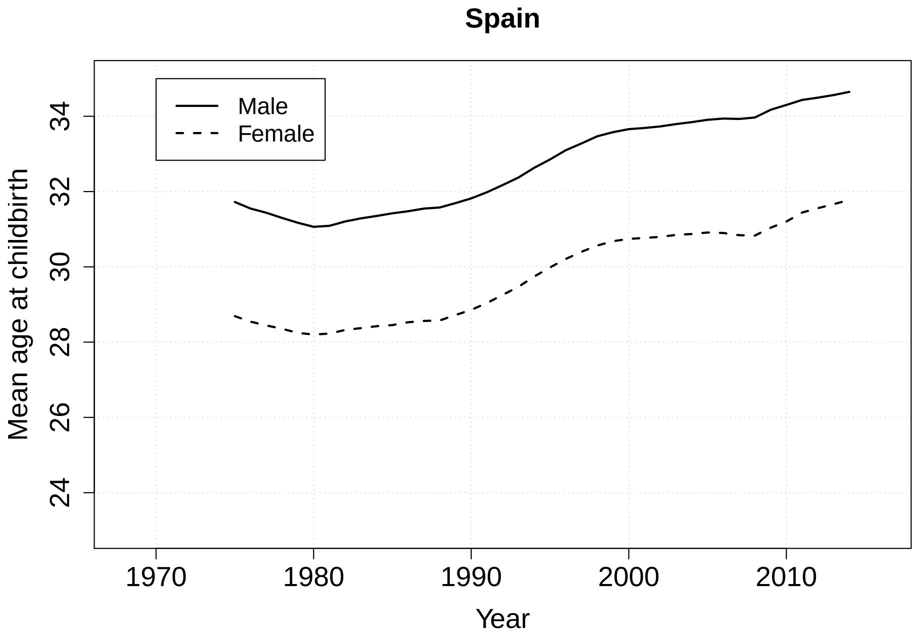

Figure C16: Mean age at childbirth by gender, Spain.

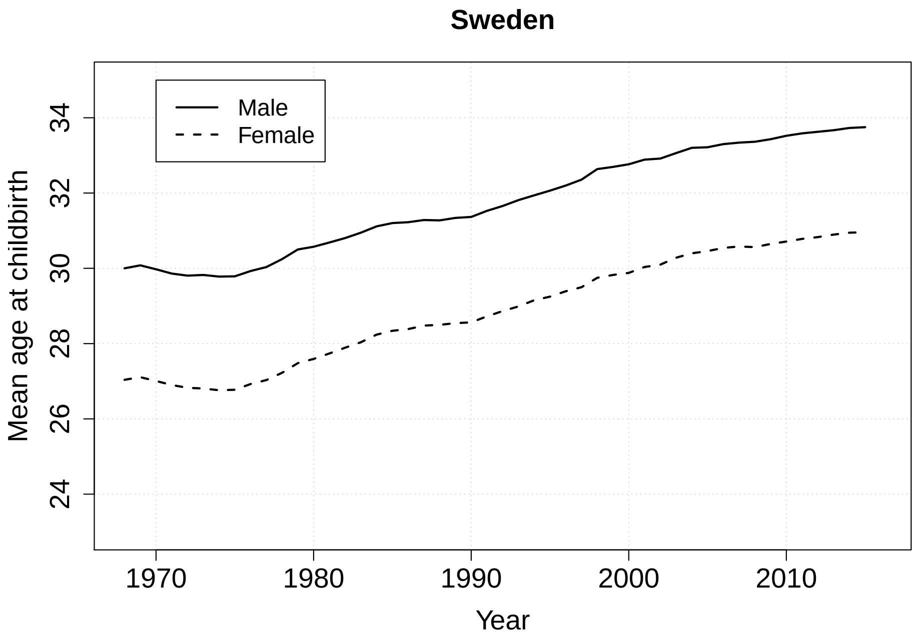

Figure C17: Mean age at childbirth by gender, Sweden.

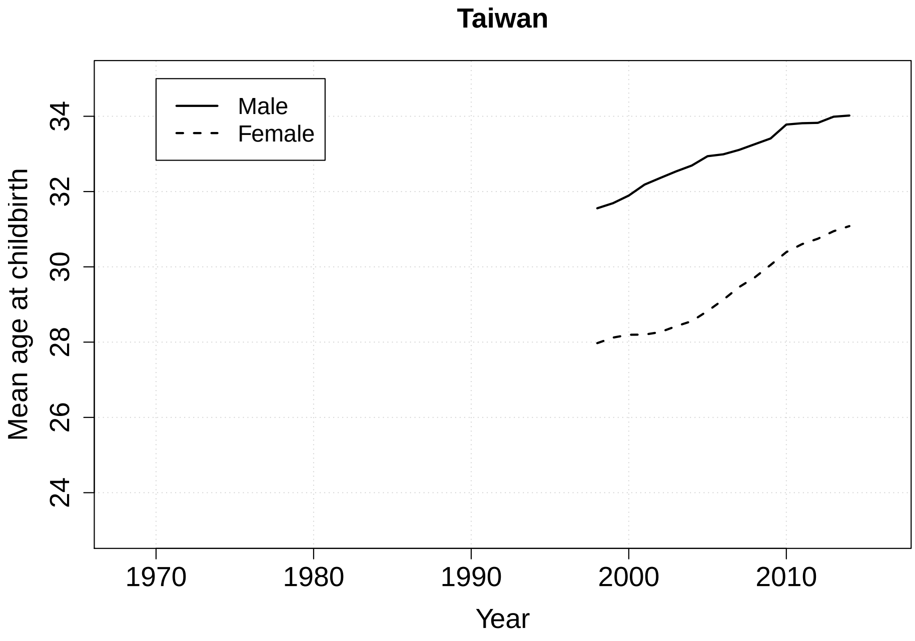

Figure C18: Mean age at childbirth by gender, Taiwan.

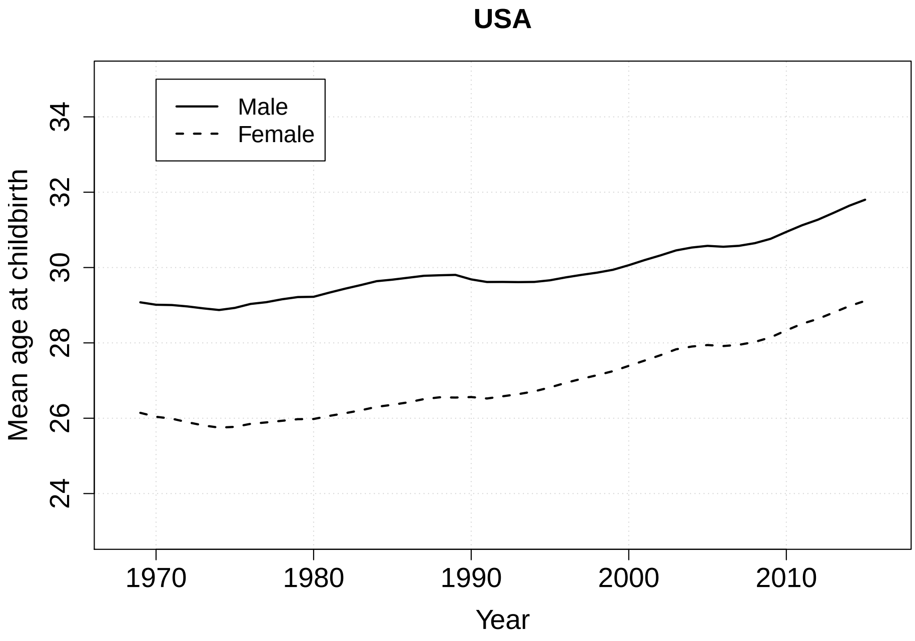

Figure C19: Mean age at childbirth by gender, USA.
